# Exploring DNA Variant Segregation Types Enables Mapping Loci for Recessive Phenotypic Suppression of Columnar Growth in Apple

**DOI:** 10.3389/fpls.2020.00692

**Published:** 2020-06-09

**Authors:** Laura Dougherty, Tuanhui Bai, Susan Brown, Kenong Xu

**Affiliations:** ^1^Horticulture Section, School of Integrative Plant Science, Cornell Agritech, Cornell University, Geneva, NY, United States; ^2^College of Horticulture, Henan Agricultural University, Zhengzhou, China

**Keywords:** DNA variants, segregation types, pooled genome sequencing, columnar suppressors, RNA-seq, co-expression gene network, MapMan Bins, *Malus*

## Abstract

Columnar apples trees, originated from a bud mutation ‘Wijcik McIntosh,’ develop a simple canopy and set fruit on spurs. These characteristics make them an important genetic resource for improvement of tree architecture. Genetic studies have uncovered that columnar growth habit is a dominant trait and is caused by a retroposon insertion that induces the expression of the neighboring gene *C*o encoding a 2OG-Fe(II) oxygenase. Here we report the genetic mapping of two loci of recessive suppressors (genes) *c2* (on Chr10) and *c3* (on Chr9) that are linked to repression of the retroposon-induced *Co* gene expression and associated columnar phenotype in 275 F_1_ seedlings, which were developed from a reciprocal cross between two columnar selections heterozygous at the *Co* locus. The mapping was accomplished by sequencing a genomic pool comprising 18 columnar seedlings and another pool of 16 standard seedlings that also carry the retroposon insertion, and by exploring DNA variants of segregation types that are informative for mapping recessive traits in apple. The informative segregation types include <**h**k × **h**k>, <**l**m × **ll**>, <**nn** × **n**p>, <**l**m × mm>, and <pp × **n**p>, where each letter denotes one of the four DNA bases and the letters in bold represent variants in relation to the reference genome. The alleles in each first and third positions are assumed in linkage with the recessive suppressors’ allele in the two parents, respectively. Using RNA-seq analysis, we further revealed that the *Co* gene together with the differentially expressed genes under loci *c2* and *c3* formed a co-expression gene-network module associated with growth habit, in which 12 MapMan Bins were enriched.

## Introduction

In the mid-20th century, the successful genetic improvement in plant architecture had led to a drastic yield increase worldwide in field crops, particularly corn, rice and wheat. The landmark accomplishment in agriculture has been known as “the Green Revolution” (Evenson and Gollin, 2003). To keep apple trees in optimal shape for fruit production in orchards, horticulturists have been improving tree pruning and training systems and developing different dwarfing rootstocks (Robinson et al., 2013). Although such efforts are effective for productivity improvement in modern orchards, apple production costs also have been increased markedly due to manual tree pruning and fruit harvest (West et al., 2012; Taylor and Granatstein, 2013). There is a strong demand for automation of labor-intensive orchard tasks, especially fruit harvest. The complex and dynamic tree canopy and variable fruit bearing sites have been the major challenges to automating fruit harvest although motorized platforms that can improve fruit harvest efficiency are available and promising prototypes of robotic fruit harvesters are being tested.

Columnar apple trees, which originally were discovered as a bud mutation from ‘McIntosh,’ called ‘Wijcik McIntosh’ in 1960s (Lapins, 1969), develop a canopy much simpler than standard apple trees do due to their reduced number of branches and vertically growing branches. Columnar cultivars usually set fruit on spurs from old woods such as the main trunk and primary limbs, requiring little pruning. These characteristics make columnar architecture an ideal fit for automation of pruning and harvesting. To take advantage of these desirable characteristics, ‘Wijcik McIntosh’ has been used in many breeding programs to develop new and improved columnar apple cultivars (Tobutt, 1984; Moriya et al., 2009). However, a major issue of existing columnar apple cultivars is their strong tendency for biennial bearing (Lauri and Lespinnasse, 1993; Petersen and Krost, 2013; Otto et al., 2014), while some studies observed that approximately 5% of columnar progeny show regular annual bearing in breeding populations, indicating columnar and biennial bearing are not always linked (Blazek, 2013; Vávra et al., 2017).

Columnar growth habit has been a major subject in apple genetic studies. An early investigation reported that the columnar growth habit was controlled by a dominant gene, called *Co* (Lapins, 1976). The *Co* locus was mapped to linkage group 10 in many studies (Conner et al., 1997, 1998; Hemmat et al., 1997; Kim et al., 2003; Tian et al., 2005; Kenis and Keulemans, 2007; Zhu et al., 2007; Fernandez-Fernandez et al., 2008; Moriya et al., 2009), and was characterized in detail (Bai et al., 2012; Moriya et al., 2012; Baldi et al., 2013; Morimoto and Banno, 2015). Sequencing analyses of the *Co* locus revealed an 8.2-kb DNA insertion (a long terminal repeat retroposon) in an inter-genic region to be genetically causal for the columnar phenotype, as the insertion is not present in ‘McIntosh’ while present in ‘Wijcik McIntosh’ (Wolters et al., 2013; Otto et al., 2014; Okada et al., 2016). Despite lacking direct interruption of any genes, the retroposon insertion increased the expression of a nearby gene encoding a 2-oxoglutarate (OG) and Fe(II)-dependent oxygenase in columnar (Wolters et al., 2013; Otto et al., 2014; Okada et al., 2016), which is called *Co* in this study. The expression of the *Co* gene was specific to root in standard apples, suggesting its expression in shoot and leaves in columnar apples is ectopic (Wada et al., 2018). Moreover, transgenic apples over-expressing the *Co* gene reduced internode length (Okada et al., 2016). These lines of evidence support that the retroposon induced ectopic expression of the 2OG-Fe(II) oxygenase encoding gene *Co* in shoots and leaves is biologically responsible for the columnar phenotype.

Despite these advances in the genetic and biological factors underlying the *Co* locus, our current understanding of the genetic control of columnar growth habit remains incomplete. This is because columnar progenies often are observed less than expected in breeding populations segregating for the trait, suggesting there are modifier genes involved (Lapins, 1976; Hemmat et al., 1997; Meulenbroek et al., 1998; Kenis and Keulemans, 2007). In the present study, we observed that among the 208 F_1_ individuals carrying the retroposon insertion in crosses between two columnar selections heterozygous at the *Co* locus, 67, 51, and 30 showed standard growth habit respectively in 2-, 4-, and 8-year-old trees, indicating there are age-dependent recessive suppressors (genes) that can suppress columnar phenotype. To identify the columnar suppressors, we explored and identified DNA variants of segregation types suitable for mapping recessive traits in apple by pooled genome sequencing, an adaptation from a previous approach developed for mapping dominant traits (Dougherty et al., 2018). We identified two recessive loci, designated *c2* and *c3* on chromosomes 10 and 9, respectively, which have a significant effect on suppressing the columnar phenotype in an age-dependable manner (more significant in young trees than in aged trees). Using RNA-seq analysis, we further revealed that suppressed columnar phenotype is coupled with a strong expression repression of the *Co* gene, which together with the differentially expressed genes (DEGs) under *c2* and *c3* formed a co-expression gene-network module highly associated with growth habit. Overall, this study represents an important first step toward revealing the identity of the causal genes under *c2* and *c3*, which would greatly increase our understanding of the genetic network responsible for columnar growth habit.

## Materials and Methods

### Plant Materials and Growth Habit Evaluation

**FIGURE 1:**
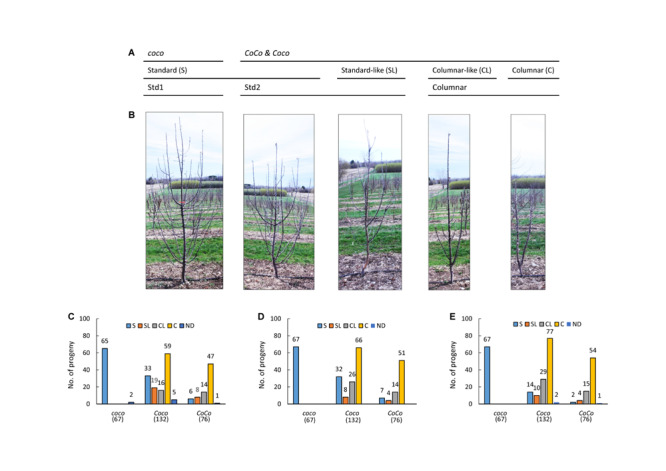
Growth habit evaluation. **(A)** Diagram of the genotypic and phenotypic relationships among Std1, Std2 and columnar. **(B)** Representative trees of standard (S), standard-like (SL), columnar (C) (developed from two independent buds) and columnar –like (CL) growth habits. The pictures were taken from 3-year-old budded trees on rootstock B118. **(C–E)** Observed genotypes at the *Co* locus and their counts in C, CL, S and SL in 2009 **(C)**, 2011 **(D)**, and 2015 **(E)**. ND, not determined.

The mapping populations were derived from a cross between NY123 (*Coco*) and NY317 (*Coco*) and its reciprocal cross, comprising 246 and 29 (275 in total) F_1_ seedling trees, respectively. Since the reciprocal cross’ contribution accounted for only 10.5% (29/275), its maternal and paternal effect was ignored. Both parents are advanced breeding selections with columnar growth habit inherited from ‘Wijcik McIntosh.’ The crosses were made in 2007 and the seedling trees were planted in spring 2008 in a Cornell University orchard in Geneva, New York. The orchard was managed with minimal pruning. The growth habit of both progenies was visually evaluated in 2009, 2011, and 2015 based on thickness of stem, number and crotch angle of lateral branches on the main axis and internode length as described previously (Bai et al., 2012). Since columnar trees usually have a thicker main stem characterized by similar diameter at the tip and the base, fewer lateral branches with narrower crotch angles, and shorter internodes, the seedling trees were first grouped into two categories: columnar and standard. Next, they were evaluated again based on these characters, dividing each category into two groups, which were scored as “1” for columnar (C) and “2” for columnar-like (CL) in category columnar, and “3” for standard-like (SL) and “4” for standard (S) in category standard for subsequent analysis (Figures 1A,B). Columnar-like is a group of columnar that are much taller and/or have a few more branches than a typical columnar, whereas standard-like is a group of standard that have relatively narrower branch angles and/or fewer lateral branches than a typical standard (Figure 1B). Some standard and all standard-like seedlings turned out to have a columnar genotype *CoCo* or *Coco*, which were called stardard2 or Std2 to differentiate from the standard seedlings of genotype *coco*, called Std1 (Figures 1A,B). Growth habit scores of the eight seedlings not determined in 2009 due to small plant size were inferred from their scores in 2011, including five columnar seedlings of genotype *Coco* and three standard seedlings of *coco* (2) and *CoCo* (1). In 2015, two *Coco* seedlings and one *CoCo* seedling died and were missing data.

Genotyping of the *Co* locus was conducted initially with DNA markers SCAR682, EMPc105, 18470-26732, C7629-22009 and HI01a03 in the *Co* region (Bai et al., 2012) and later with 29f1/JWI1r, which detects the retroposon insertion (Wolters et al., 2013).

### Pooled Genome Sequencing Analysis

Genomic DNA samples from 18 columnar and 16 Std2 plants (Supplementary Figure S1 and Supplementary Table S1) were isolated in 2015 again and pooled equally with 500 ng from each progeny to construct a columnar pool and a Std2 pool, respectively. Sequencing and data analysis of the two genomic pools were conducted similarly as previously described (Dougherty et al., 2018). Briefly, the two pooled genomic DNA libraries of target insert size of 500 bp were constructed using NEBNext Ultra DNA Library Prep Kit for Illumina (New England Biolabs, Ipswich, MA, United States), and then paired-end (2 × 151 bp) sequenced on an Illumina Nextseq500 platform at the Genomics Facility of Cornell University (Ithaca, New York, United States) (NCBI SRA accession PRJNA547442). Software CLC Genomics Workbench (v11.0, CLCBio, Cambridge, MA, United States) was employed to map the sequence reads against the apple reference genome (Daccord et al., 2017) for each genomic pool (Supplementary Table S2). The reads mapping parameters were set as the following: minimum length fraction 0.8 and the minimum similarity 0.98, similar to those described earlier (Bai et al., 2014).

DNA variants were called for each pool using the fixed ploidy (2×) variant detection tool of CLC Genomics Workbench, which automatically calculates read coverage and variant frequency. A minimum coverage of ten and a minimum count of two for variant-carrying reads were used initially. Next, the variants were filtered to remove those that are reference alleles, non-single nucleotide variants (SNVs), or with reads coverage lower than 20× or greater than 200×, resulting in one set of SNVs specific to pool Std2, another specific to columnar, and the third common to both pools (Supplementary Figure S1).

### Inferring Informative Variant Segregation Types for Mapping Recessive Trait

The strategy for inferring variant segregation types informative for mapping a dominant trait described previously (Dougherty et al., 2018) was followed. However, it was adapted to recessive Std2, which was necessary due to recessive inheritance (see also section “Discussion”). The task here is to identify variants that not only situate with high density in the genomic regions targeted by phenotypic pooling, but also differ widely between pools Std2 and columnar in variant allele frequency (AF) due to their physical linkage to the causal genes and due to their segregation types. To facilitate the identification of informative variants for mapping, the SNVs (1,997,962) common to both pools were grouped into heterozygotes (15% ≤ AF < 85%) and homozygotes (AF ≥ 85%). Combining their allele frequency with source pools, the SNVs were further divided into four groups (Supplementary Table S3): (1) homozygotes in Std2 and heterozygotes in columnar (Ho-Std2/He-Col); (2) heterozygotes in both Std2 and columnar (He-Std2/He-Col); (3) heterozygotes in Std2 and homozygotes in columnar (He-Std2/Ho-Col); and (4) homozygotes in Std2 and homozygotes in columnar (Ho-Std2/Ho-Col).

In a typical bi-parental cross in apple, the segregation of SNVs could be determined by up to six possible segregation types. They include <ab × cd>, <ef × eg>, <hk × hk>, <lm × ll>, and <nn × np>, and < qq × qq>, where each letter stands for one of the four DNA bases (A, C, G, and T) in SNVs (Dougherty et al., 2018). Among them, variants of the segregation type <qq × qq> clearly are non-informative, whereas those in the other five types are considered informative for mapping Std2. However, variants of segregation types <ab × cd> and <ef × eg> are rare in the genome due to the involvement of four or three DNA bases, thereby these were not further pursued. The remaining variants of segregation types <hk × hk>, <lm × ll>, and <nn × np>, which involve only two nucleotides, are more abundant and suitable for mapping. When the SNVs linkage to the Std2 allele (i.e., haplotype) is considered, the three suitable segregation types could be expressed with at least 12 derivatives (Supplementary Table S3).

Examining the allele frequency of SNVs under each of the 12 possible segregation types under the model of one- or two-recessive genes revealed five variant segregation types potentially informative and suitable for mapping recessive Std2. They include <**h**k × **h**k>, <**l**m × **ll**>, <**nn** × **n**p>, <**l**m × mm>, and <pp × **n**p>, named segregation types A, B, C, D, and E, respectively. Here the letters in bold represent SNVs in relation to the reference genome, and the alleles in each first and third positions are assumed to be in linkage with the recessive Std2 alleles in the seed and pollen parents, respectively (Supplementary Figure S2 and Supplementary Table S3). The other seven segregation types were not informative for mapping recessive Std2 due to either an equal SNV allele frequency in both pools or a negative SNV allele frequency margin (informative for mapping the allele dominant over Std2) between pools Std2 and columnar.

Variants under segregation types A, B, and C were inferred to be homozygous in pool Std2 and heterozygous in columnar (Ho-Std2/He-Col). Considering cases involving one- or two- recessive genes, the variant allele frequency in pool columnar would be 33.3 and 46.7% for type A (<**h**k × **h**k>), and 66.7 and 73.3% for both types B (<**l**m × **ll** >) and C (<**nn** × **n**p>), respectively. The variant allele frequency directional (positive) difference (AFDD) between pools Std2 and columnar would be 66.7 and 53.3 percentage points for type A, and 33.3 and 26.7 percentage points for both types B and C, respectively (Supplementary Figure S2 and Supplementary Table S3).

Similarly, variants under segregation types D (<**l**m × mm>) and E (<pp × **n**p>) were inferred to be heterozygous in both pools, but different in their variant allele frequency. Under the model of one- or two- recessive genes, the allele frequency in pools Std2 and columnar would be 50.0 and 16.7%, and 50.0 and 23.3%, corresponding to AFDD 33.3 and 26.7 percentage points, respectively (Supplementary Figure S2 and Supplementary Table S3).

These analyses confirmed that only the variants common to both pools could be informative for mapping the recessive Std2 trait while none of the pool specific variants would be informative. Within the pool common variants, only those that are in groups Ho-Std2/He-Col and He-Std2/He-Col are useful potentially (Supplementary Figures S1, S2 and Supplementary Table S3).

### Identification of Informative SNVs for Mapping Recessive Std2

The allele frequency directional (positive) difference and density (AFDDD) mapping approach, which explores DNA variants that are common to both pools as previously described (Dougherty et al., 2018), was adapted to mapping recessive Std2. This was accomplished by identifying informative SNVs based on the expected variant allele frequencies in each pool and their expected directional (positive) differential margins between pools Std2 and columnar under each of the five informative segregation types (Supplementary Figures S1, S2 and Supplementary Tables S3, S4). For segregation type A (<**h**k × **h**k>), the 70,522 SNVs in group Ho-Std2/He-Col were subjected to two filters: (1) Variant allele frequency ≥ 85% in pool Std2. (2) The AFDD ≥ 43.3 percentage points between pools Std2 and columnar to cover the expected AFDD 66.7 and 53.3 (percentage points) under the model of one- and two-recessive genes, respectively (Supplementary Table S4). For segregation types B (<**l**m × **ll**>) and C (<**nn** × **n**p>), the 70,522 SNVs were filtered similarly as described above. However, the AFDD was limited to a range from 16.7 to 43.3 percentage points between pools Std2 and columnar (Supplementary Table S4). For segregation types D (<**l**m × mm>) and E (<pp × **n**p>), the 1,636,085 variants that were in group He-Std2/He-Col were filtered with two filters: (1) the DNA variant AF ranges from 35 to 65% in the Std2 pool, close to their estimated mean 50%. (2) The AFDD is restricted from 16.7 to 43.3 percentage points between the Std2 and columnar pools (Supplementary Table S4).

Such resultant informative variants (SNVs), either combined or individually according to their segregation types, were then plotted along the reference genome and visualized using a total number of variants in 1-Mb sliding windows. Genomic regions of variant density significantly higher than the genome average in standard score (*z*) test were consider putatively linked to the recessive trait Std2. The *z*-test was conducted in MS-Excel or R if the *p*-values were lower than 1.0E-7, and the cutoff *p*-value (two-tailed confidence level) is -log_10_p(z) (called LODz for convenience) > 2.5.

### Development and Analysis of DNA Markers in Genomic Regions Putatively Linked to Std2

As a validation step, independent DNA markers were used to confirm the putative genetic linkage between trait Std2 and the *c2, c3*, and other positive regions identified by AFDDD mapping in pooled genome sequencing analysis. Existing SSR markers in these regions were applied first. If necessary, new SSRs would be developed from the apple reference genome as described earlier (Xu et al., 2012). Polyacrylamide gel electrophoresis of SSR markers were conducted as detailed previously (Wang et al., 2012). In addition, high-resolution melting (HRM) markers were developed by targeting SNVs of segregation type A-C in coding regions of genes. Analysis of HRM markers was performed using a CFX96 Real-Time PCR Detection System in combination with Precision Melt Super Mix and software packages CFX Maestro and High Resolution Melting Analysis following the manufacturer’s instruction (Bio-Rad, Hercules, CA, United States). The SSR and HRM marker primer sequences and their approximate physical locations in the reference genome were provided (Supplementary Table S5).

### RNA-seq Analysis

Twelve *F*_1_ seedlings evenly representing phenotypes columnar (4), Std1 (4), and Std2 (4) were budded onto apple rootstock B118 in 2015 and planted in the orchard in spring 2016 (Figures 1A,B). In June 2017, the actively growing shoot apex tissues (leaves removed) were collected and flash frozen in liquid nitrogen for RNA isolation. The shoot apex tissues were ground in liquid nitrogen and total RNA was isolated as previously described (Meisel et al., 2005). The RNA samples were treated with DNase I (amplification grade, Invitrogen, Carlsbad, CA, United States) and cleaned with RNeasy MinElute Clean up Kit (Qiagen, Hilden, Germany). RNA concentration and quality were determined using NanoDrop 1000 (Thermo Fisher Scientific, Waltham, MA, United States) and assays on a 1.0% agarose gel.

mRNA was isolated from total RNA using NEBNext Poly(A) mRNA Magnetic Isolation Module and was used to construct RNA-seq libraries with NEBNext Ultra Directional RNA Library Prep Kit for Illumina (New England Biolabs, Ipswich, MA, United States) as previously described with slight modification (Bai et al., 2014). Briefly, libraries were size-selected for 350–500 bp and were multiplexed in equal amount for single end 76-base sequencing by NextSeq 500 (Illumina, San Diego, CA, United States) at the Cornell University Biotechnology Resource Center (Ithaca, NY, United States). Illumina sequencing of the 12 RNA-seq libraries generated 12 FASTQ files of sequences (NCBI SRA accession PRJNA547442) with 409.9 million raw reads in total (Supplementary Table S6). Cleaning-up of the raw reads, including removal of adaptors, rRNA contaminations and low quality and/or short reads, was conducted similarly as described previously (Bai et al., 2015). The resultant high quality reads were mapped to the apple reference genome (Daccord et al., 2017) using CLC Genomics Workbench 11.0 (Minimum similarity fraction: 0.98, minimum length fraction: 0.8, and maximum number of hits: 10). Gene expression levels were calculated and represented by reads per kilobase of exon model per million mapped reads (RPKM) (Mortazavi et al., 2008). Genes were considered expressed when their mean RPKM > 0.25 in any of the three sample groups (columnar, Std1 and Std2). DEGs were defined as those of RPKM folder change ≥ 1.5 and *P*_FDR_ ≤ 0.05 among the three groups, in which each of the four F_1_ individuals was considered a biological replicate.

### Weighted Gene Co-expression Network Analysis (WGCNA)

DEGs among phenotype groups columnar, Std1 and Std2 were analyzed using WGCNA, an R package (Langfelder and Horvath, 2008) to identify co-expression gene network modules associated with growth habit. The significance cutoff is *p* < 0.001. Relevant parameters were set similarly as described previously (Bai et al., 2015). Visualization of the most significant WGCNA module was accomplished using Cytoscape 3.6 (Saito et al., 2012). Analyzing the network was conducted using a Cytoscape plugin Network Analyzer (Assenov et al., 2008).

### MapMan Annotation and Gene Enrichment Analysis

Annotations of the reference genome with MapMan Bins was assisted with Mercator (Lohse et al., 2014), resulting in assigning a MapMan bin to 45,116 genes. Gene enrichment analysis was performed for the WGCNA module that shows the highest correlation with tree growth habit using the hypergeometric annotation test tool available in CLC Genomics Workbench, which is similar to the unconditional GOstats test (Falcon and Gentleman, 2007). For declaration of significant enrichment, the cutoff is *P*_FDR_ < 0.05.

### Quantitative (q) RT-PCR

The same set of plant samples taken in June 2017 for RNA-seq were used, and another set taken in June 2018 was used to repeat the *Co* gene expression analysis. Two microgram of total RNA was used in reverse transcription reactions using the iScript^TM^ cDNA Synthesis Kit (Bio-Rad, Hercules, CA, United States) to obtain the first strain of cDNA, and then used as templates for qRT-PCR analysis. The qRT-PCR reactions were performed with three technical replicates using iTaq^TM^ Universal SYBR^®^ Green Supermix on a CFX96 Real-Time PCR Detection System according to manufacturer’s protocol. An apple actin encoding gene (MD01G1001600) was used as a reference gene. The expression levels of target genes were quantified based on the normalized expression (ΔΔCq) of the reference gene using the Bio-Rad CFX Maestro software. qRT-PCR primers were designed for eight genes expressed in the libraries (Supplementary Table S5). The normalized gene expression from qRT-PCR was compared to the RNA seq gene expression in RPKM.

### Statistical Analysis

Analysis of variance (ANOVA) and regression analysis were conducted using JMP Pro12 (SAS, Cary, NC, United States).

## Results

### Segregation of Columnar and Standard Phenotypes

Genotyping with the *Co* linked markers confirmed a normal 1:2:1 (76 *CoCo*:132 *Coco*:67 *coco*) segregation at the *Co* locus in the 275 *F*_1_ plants (χ^2^ = 1.029, *p* = 0.5978). However, evaluation of their growth habit in 2009, 2011, and 2015 revealed that the expected 3:1 (columnar: standard) segregation was significantly distorted (χ^2^ = 82.46, *p* = 2.2E-16 in 2009; χ^2^ = 47.04, *p* = 7.0E-12 in 2011; χ^2^ = 16.49, *p* = 4.9E-5 in 2015) for columnar (C) and columnar-like (CL) vs. standard (S) and standard-like (SL) due to excessive S/SL individuals. Inspecting the segregation data (Figures 1C–E) indicated the following: (1) All 67 seedlings of genotype *coco* were consistently standard. (2) In the 208 seedlings of genotypes *CoCo* and *Coco*, 141 consistently exhibited C/CL as expected. (3) The remaining 67 individuals of genotypes *CoCo* and *Coco* were unexpectedly scored as standard and standard-like in 2009, and were progressively reduced to 51 in 2011, and 30 in 2015. That is to say that 37 of the 67 S/SL individuals in 2009 progressively returned to C/CL while the other 30 remained unchanged (Supplementary Figure S1).

These observations suggested that the presence of these 30–67 S/SL individuals of genotypes *CoCo* and *Coco* directly caused the phenotypic segregation distortion. For convenience, the standard phenotype associated with genotype *coco* is called standard1 (Std1), and that with *CoCo* and *Coco* called standard2 (Std2) (Figures 1A,B). Since the Std2 individuals accounted for 14.4 to 32.2 percent in the *CoCo* and *Coco* genotype groups, there are age-dependent recessive suppressors (genes) that can suppress the columnar phenotype more effectively in young trees than in older trees.

### Pooled Genome Sequencing Based AFDDD Mapping of the Recessive Suppressors of Columnar

Sequencing generated 166.5 million and 259.1 million reads in pools Std2 and columnar, covering the apple reference genome (Daccord et al., 2017) by 35.4× and 55.1×, respectively (Supplementary Figure S1 and Supplementary Table S2). Removing low quality reads and bases resulted in 163.5 million 125.0-bp cleaned reads for pool Std2 and 253.0 million 125.8-bp clean reads for pool columnar (Supplementary Table S2). Reads mapping against the reference genome mapped 127.5 million (78.0%) of the clean reads in pool Std2 and 195.1 million (77.1%) in columnar, covering the genome by 22.5× and 34.6×, respectively (Supplementary Table S2).

Detection of DNA variants reported 56,571 SNVs specific to pool columnar, 14,078 specific to Std2, and 1,997,962 common to both pools (Supplementary Figure S1). Among the variants common to both pools, 70,522 (3.5%) were homozygotes in Std2 and heterozygotes in columnar (Ho-Std2/He-Col), 1,636,085 (81.9%) were heterozygotes in both Std2 and columnar pools (He-Std2/He-Col), 39,075 (2.0%) were He-Std2/Ho-Col, and 252,280 (12.6%) were Ho-Std2/Ho-Col (Supplementary Table S3). Notably, a considerable fraction (14.6–16.2%) of the SNVs common to both pools had a variant allele frequency (AF) ≥ 85% (close to be homozygous) (Supplementary Figures S3A,B) while 90.6% of the SNVs specific to pool Std2 and 94.0% of the SNVs specific to pool columnar had a variant AF ≤ 45% (Supplementary Figures S3C,D).

Since the pool specific SNVs were non-informative for mapping Std2, and the informative SNVs were characterized with a specific range of allele frequencies in the two pools (Supplementary Figure S2 and Supplementary Tables S3, S4), we identified 7,642 informative variants under segregation type A, 40,166 under types B and C, and 70,230 under types D and E (Supplementary Table S4).

**FIGURE 2:**
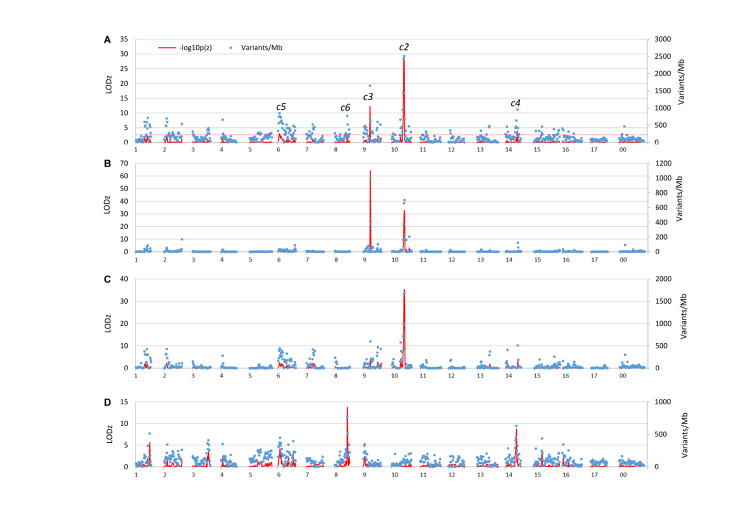
Variants allele frequency (AF) directional difference (AFDD) and density (AFDDD) mapping of columnar recessive suppressors (genes) using the five informative segregation types of 118,038 SNVs **(A)**, type A <**h**k × **h**k> of 7,642 SNVs **(B)**, types B-C <**l**m × **ll**> and <**nn** × **n**p> of 40,166 SNVs **(C)**, and types D-E <pp × **n**p> and <**l**m × mm> of 70,230 SNVs **(D)**. The numbers on *X*-axis represent apple chromosomes. The line in red dashes indicates the cutoff LODz [−log_10_p(z)] 2.5 in *z*-score test of AFDDD in **(A)**.

**FIGURE 3:**
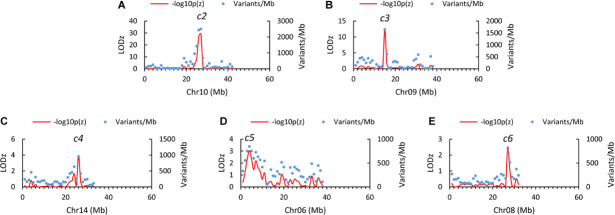
Close-up views of the genomic regions mapped by AFDDD mapping for putative columnar recessive suppressors *c2* **(A)**, *c3* **(B)**, *c4* **(C)**, *c5* **(D)**, and *c6* **(E)**. Note that the *c2* region is close to the *Co* gene, which located at 28.0th Mb on chromosome 10.

Examining the genome distribution of the three sets of informative SNVs collectively (118,038 in total) revealed five genomic regions of significantly higher variant density than the genome average, tentatively named *c2, c3, c4, c5*, and *c6*, respectively (Figure 2A). The peaks of *c2* and *c3* are located at the 27th (26–27) Mb on chromosome 10 (LODz = 29.2), and the 15th Mb on chromosome 9 (LODz = 12.2), respectively (Figure 3). The peak locations of *c4, c5*, and *c6* were at 26th Mb on chromosome 14 (LODz = 3.92), the 4th Mb on chromosome 6 (LODz = 3.04), and the 27th Mb on chromosome 8 (LODz = 2.51), respectively (Figure 3). The *c2* and *c3* regions likely represent the major loci relevant for phenotype Std2.

To see if the five variant segregation types contribute differently to the identified regions, the genome distribution of the three sets of variants were examined independently (Figures 2B–D). The results demonstrated that the two major loci *c2* and *c3* were determined by SNVs of segregation types A, B, and C, *c5* and *c6* by those of types D and E, and *c4* by all segregation types. Therefore, segregation types A, B, and C appeared to be more useful than types D and E in this study.

### Confirmation of the Mapping of Columnar Suppressors

Identification of loci *c2* to *c6* indicated putative mapping of the recessive columnar suppressors. For confirmation, 13 existing and newly developed SSR and HRM markers in these regions were analyzed in the populations (Supplementary Table S5). To maximize the confirmation, the 2009 phenotypic data were used. Based on the marker-trait linkage analysis in the 208 seedlings of genotypes *CoCo* and *Coco*, loci *c4 to c6* were not confirmed (data not shown) while loci *c2* and *c3* were confirmed and described below.

The *c2* locus was represented by marker AU223548, which is located at the 26.35th Mb on chromosome 10, roughly 1.65 Mb upstream of the *Co* gene (MD10G1185400) encoding a 2OG-Fe(II) oxygenase (Wolters et al., 2013; Otto et al., 2014; Okada et al., 2016). The observed recombination rates between the two loci were 0.079 in NY123 and 0.113 in NY317, suggesting a moderate linkage between them. Since the suppression effect of columnar could not be detected in the *coco* group, the genetic effect of *c2* was investigated only in genotype groups *CoCo* and *Coco*. The null hypothesis is that the *c2* and *C2* alleles from a given parent may segregate differently between genotype groups *coco* and *CoCo/Coco* due to linkage, but would segregate similarly across *CoCo* and *Coco* irrespective of their phenotypes columnar and Std2. A significant segregation distortion from what is expected in phenotype group Std2 and/or columnar would indicate a linkage between *c2* and Std2. The parental recessive *c2* alleles were defined as those whose frequencies were increased significantly in Std2.

Chi-square analysis of marker AU223548 genotypes indicated that the segregation of parental alleles was significantly distorted in Std2 (*p* = 0.0011 in NY123; *p* = 6.40E-6 in NY317) (Supplementary Figures S4A,B) and columnar (*p* = 0.0256 in NY123; *p* = 0.0021 in NY317) (Supplementary Figures S4D,E). Consequently, the *c2c2* individuals were significantly more frequent (*p* = 6.28E-10) than what was expected in Std2 while the *C2C2* individuals were significantly more (*p* = 1.17E-6) in columnar (Supplementary Figures S4C,F). These observations strongly supported the genetic mapping of locus *c2*. Examining the relationships between loci *Co* and *c2* revealed that NY123 and NY317 are of genotypes *Coc2coC2* and *CoC2coc2* (the underlines denote haplotype), respectively. Therefore, the recessive *c2* allele is linked to the dominant *Co* allele in coupling phase in NY123, and in repulsion phase in NY317.

The *c3* locus was confirmed by marker Hi05e07, which is located at the 14.3th Mb on chromosome 9. The marker segregation was distorted for the NY317 alleles in Std2 (*p* = 0.0055) and columnar (*p* = 0.0546) (Supplementary Figures S4H,K), but was normal for the NY123 alleles (*p* = 0.1894 in Std2 and 0.3641 in columnar) (Supplementary Figures S4G,J). This suggested that NY317 and NY123 are heterozygous and homozygous at the *c3* locus, respectively. The genotype of NY123, therefore, is inferred as *Coc2coC2 c3c3*, and that of NY317 as *CoC2coc2 C3c3*. The distorted segregation of NY317 alleles led to a significant increase in the number of *c3c3* individuals in Std2 (*p* = 0.0189) although the increase for the *C3C3* progenies were not significant (*p* = 0.1232) in columnar (Supplementary Figures S4I,L).

**FIGURE 4:**
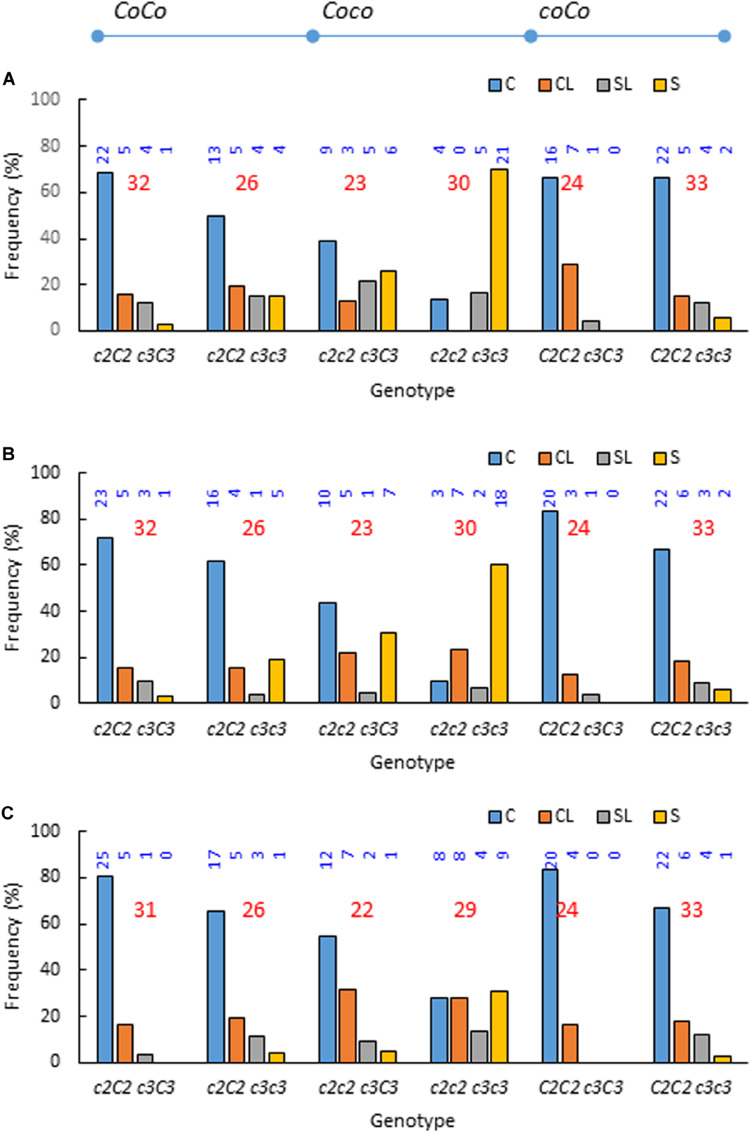
Phenotypic frequencies in each of the six possible genotypes observed in years 2009 **(A)**, 2011 **(B)**, and 2015 **(C)** when recombinants between *Co* and *c2* were excluded due to limited number for meaningful comparisons. The six genotypes are *Coc2CoC2 c3C3*, *Coc2CoC2 c3c3*, *Coc2coc2 c3C3*, *Coc2coc2 c3c3*, *coC2CoC2 c3C3*, and *coC2CoC2 c3c3*, i.e., *c2C2 c3C3*, *c2C2 c3c3*, *c2c2 c3C3*, *c2c2 c3c3*, *C2C2 c3C3* and *C2C2 c3c3* if omitting *Co* alleles. The underline denotes haplotypes, and the genotypes are signified with alleles from NY123 and NY317 in order. C, columnar; CL, columnar-like; S, standard; SL, standard like. The numbers in blue and red indicate the individual and total numbers of progenies in each of the six genotypes, respectively.

Investigating the phenotypic frequencies in each of the six possible genotype groups (three genotypes *Coc2CoC2,Coc2coc2,* and *coC2CoC2* at *c2* by two genotypes *c3c3* and *c3C3* at *c3*) further supported the association between phenotype Std2 and recessive genotypes *c2c2* and *c3c3* (Figure 4). The six genotypes are *c2C2 c3C3*, *c2C2 c3c3*, *c2c2 c3C3*, *c2c2 c3c3*, *C2C2 c3C3*, and *C2C2 c3c3* when omitting the *Co* alleles. In 2009, the frequencies of Std2 were high in double recessive carriers *c2c2 c3c3* (0.867), medium in single recessive *c2c2 c3C3* (0.478), *c2C2 c3c3* (0.308) *and C2C2 c3c3* (0.182), and low in non-recessive *c2C2 c3C3* (0.156) and *C2C2 c3C3* (0.042). The overall frequency of Std2 was reduced by tree age; however, the trend remained (Figure 4). By 2015, the frequencies of Std2 in double recessive, single recessive and non-recessive were 0.448, 0.136–0.154, and 0–0.032, respectively. These observations also suggested that the penetrance of phenotype Std2 was incomplete even in double recessive *c2c2 c3c3*, ranging from 0.867 in 2009 to 0.448 in 2015.

### Genetic Effect of *c2* and *c3* on Suppression of Columnar

To quantify the genetic effect of *c2* (AU223548) and *c3* (Hi05e07) on suppression of the columnar phenotype in genotype groups *CoCo* and *Coco*, regression analyses were conducted by assigning the phenotypes columnar, columnar like, standard like and standard with scores 1, 2, 3, and 4, respectively. The results revealed that locus *c2* accounted for 19.2% of the phenotypic variation in 2009, 16.2% in 2011, and 10.0% in 2015, greater than what was estimated for locus *c3*, which are 8.1, 6.9, and 7.0%, respectively, and the two loci combined explained 25.7, 22.2, and 15.7% of the population variance in 2009, 2011, and 2015, respectively (Table 1). The regression model fit *p*-values ranged from 4.93E-12 to 6.68E-04, which were all significant (Table 1). Overall, *c2* seemed to play a much greater role in suppression of columnar in younger trees than in older trees while the influence of *c3* was constant relatively.

**TABLE 1:** Regression analyses of the effect of loci *c2* and *c3* on repression of columnar phenotype.

Loci	2009			2011			2015		
	*r*^2^	*p*	*n*	*r*^2^	*p*	*n*	*r*^2^	*p*	*n*
*c2* (AU223548)	0.1921	6.02E-10	202	0.1619	1.37E-08	208	0.0997	2.48E-05	205
c3 (Hi05e07)	0.0806	2.34E-04	202	0.0692	6.68E-04	207	0.0702	6.66E-04	204
c2 and c3	0.2574	4.93E-12	202	0.2219	2.32E-10	207	0.1571	6.88E-07	204

*P (for model fit) is determined by ANOVA.*

### Transcriptomic Characterization of Main Shoot Apex in Columnar, Standard1 and Standard2

**FIGURE 5:**
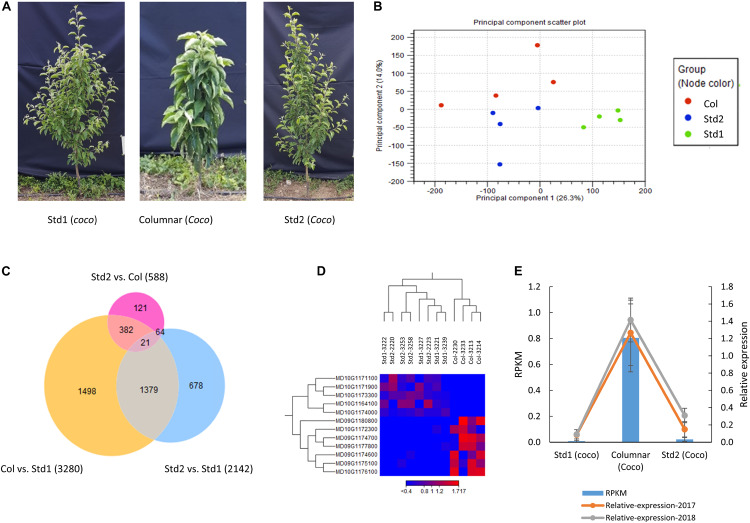
Differentially expressed genes (DEGs) in actively growing main shoot apex tissues among the columnar, Std1 and Std2 progenies. **(A)** Photos of typical trees sampled for RNA-seq analysis. The trees were 2-year-old (in 2017) budded on rootstock B118, and their main shoot apex tissues were taken for RNA isolation. **(B)** Principal component analyses of 12 RNA-seq samples of columnar (Col), standard1 (Std1) and Standard2 (Std2) growth habits. **(C)** Venn Diagram analysis of the DEGs among the three phenotypes. The numbers in parenthesis indicate the sum of DEGs in each comparison. **(D)** DEGs in the genomic regions of *c2* and *c3*. **(E)** Expression repression of the *Co* gene in Std2. Relative expression levels were determined by qRT-PCR from samples (*n* = 3 × 4) taken in 2017 and 2018, respectively. RPKM, reads per kilobase of transcript per million mapped reads.

An RNA-seq analysis was conducted on actively growing main shoot apex tissues from four columnar, four Std1 and four Std2 seedlings grafted on rootstock B118 (Figure 5A and Supplementary Table S6) to investigate what genes were expressed differentially genome-wide and locally in the *c2* and *c3* regions and how the *Co* gene (MD10G1185400) was repressed in the three groups. After removal of low quality reads and rRNA contaminations, 334.2 million clean reads (76 bp) in total were obtained from the 12 libraries, and 279.3 million (83.6%) of them were mapped to the apple reference genome (Daccord et al., 2017), equivalent to 23.3 ± 9.7 (83.7 ± 1.2%) million mapped reads per sample (Supplementary Table S6). In total, there were 33,430 genes expressed (mean RPKM ≥ 0.25 in columnar, Std1 or Std2). Principle component (PC) analysis of the gene expressions revealed that samples in Std1 form a tight group while samples in Std2 and columnar appeared to form their own groups as well despite more spreading (Figure 5B). Pair-wised comparison among the three groups identified 588 DEGs between Std2 and columnar, 2142 between Std2 and Std1, and 3280 between columnar and Std1 (Figure 5C and Supplementary Table S7), suggesting Std2 resembled columnar more than Std1, consistent with the results in PC analysis (Figure 5B). Venn diagram analysis showed that there were 4,143 non-redundant DEGs among the three groups (Figure 5C).

In the 588 DEGs between Std2 and columnar, 392 (66.7%) were down regulated in Std2 while 196 (33.3%) were upregulated. In contrast, 1667 (50.8%) of the 3280 DEGs between Std1 and columnar were down regulated in Std1 while 1613 (49.2%) were upregulated (Supplementary Figure S5), suggesting that a higher proportion of the DEGs were downregulated in Std2 than in Std1.

Validation of RNA-seq based expression was conducted by qRT-PCR analysis on eight genes (Supplementary Figures S6A–H). Highly significant correlations in gene expression were observed between qRT-PCR and RNA-seq (*R*^2^ = 0.5217 to 0.9479; *p* = 7.98E-3 to 9.66E-8; *n* = 12), indicating the RNA-seq data were reliable.

### Differentially Expressed Genes Under *c2* and *c3*, and Repression of *Co* in Standard2

In the *c2* (from the 25th to 27th Mb on Chr. 10) and *c3* (from the 14th to 16th Mb on Chr. 9) peak regions (Figures 3A,B), there were 155 and 173 genes annotated, of which 109 and 101 were expressed, respectively (Supplementary Table S8). Despite the large number of annotated and expressed genes, the DEGs between columnar and Std2 were limited to seven under *c2* and five under *c3* (Figure 5D and Supplementary Table S8). Of the seven DEGs under *c2*, two genes MD10G1172300 (encoding a glutathione *S*-transferase TAU 8-like) and MD10G1176100 (Long-chain fatty alcohol dehydrogenase family protein) were downregulated in Std2, whereas the other five were upregulated, including MD10G1171100 encoding a GDSL lipase, and MD10G1171900, MD10G1173300 and MD10G1174000 of unknown function. The five DEGs under *c3* were all downregulated in Std2, including MD09G1174600, MD09G1174700 and MD09G1175100 encoding a GDSL lipase, MD09G1177800-an aldolase-type TIM barrel family protein, and MD09G1180800-a protein of unknown function (Figure 5D and Supplementary Table S8). These DEGs were considered important candidate genes as the columnar suppressors. Interestingly, four of the 12 DEGs are GDSL-like genes.

The expression of the *Co* gene (MD10G1185400) was relatively low in columnar (RPKM 0.805 ± 0.262), but clearly detectable. Surprisingly, its expression in Std2 was reduced by 27.8 fold to RPKM 0.021 ± 0.014, close to RPKM 0.008 ± 0.017 in Std1 that was virtually undetectable, suggesting a drastic repression of *Co* (Figure 5E and Supplementary Figure S6A). These expression patterns were also detected in qRT-PCR analyses using the same or similar shoot apex tissues collected in 2017 and 2018 (Figure 5E). Since the induced higher expression of *Co* by the retroposon insertion is responsible for the columnar phenotype (Wolters et al., 2013; Otto et al., 2014; Okada et al., 2016), the repression of *Co* expression may have suppressed columnar, leading to the Std2 phenotype.

To search for SNVs that could potentially lead to recessive Std2, the 155 genes annotated under *c2* and 173 under *c3* were investigated for the presence of non-synonymous mutations that are among the 47,808 SNVs under segregation types A-C (Supplementary Table S4), which are homozygous in pool Std2 (Supplementary Table S3). It was revealed that 58 and 25 expressed genes under *c2* and *c3* carry at least one non-synonymous SNV, respectively (Supplementary Table S8). Interestingly, such mutation-carrier genes include two (MD10G1164100 and MD10G1176100) of the seven DEGs under *c2*, three (MD09G1174600, MD09G1174700, and MD09G1175100) of the five DEGs under *c3*, and two non-DEGs (MD10G1165100 and MD09G1170200) that are putative transcription regulators (Supplementary Table S8).

### Identification of a *Co* Guided Co-expression Gene Network Module

**FIGURE 6:**
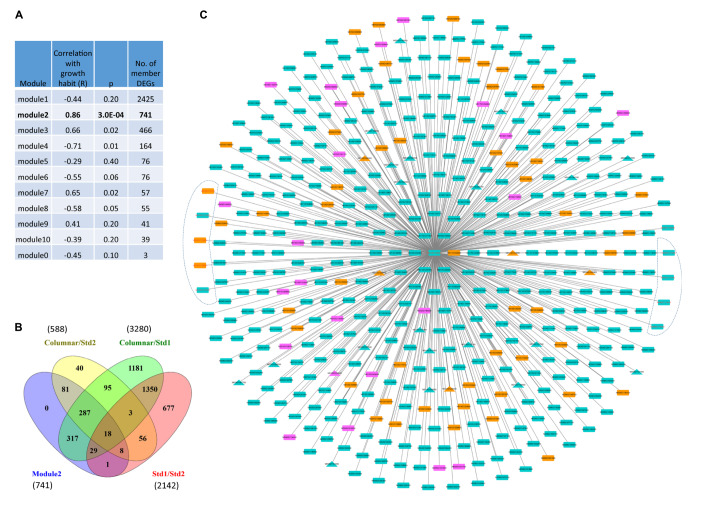
Weighted gene co-expression network analysis (WGCNA) of DEGs among progenies of phenotypes columnar, Std1, and Std2. **(A)** Correlations between WGCNA modules and tree growth habit (columnar, Std1, and Std2). **(B)** Venn Diagram analysis of the 741 member genes of WGCNA module2 associated with tree growth habit. **(C)** Primary neighbors of the *Co* gene in WGCNA module2. Orange: 57 upregulated in Std2 (in relation to columnar); Turquoise: 389 down regulated in Std2; Purple: 23 DEGs with absolute fold change ≤ 1.5. Triangle: transcriptional factors. DEGs in the *c2* and *c3* regions are indicated in an irregular shape on the left and right, respectively. Note that other edges are not shown.

Weighted gene expression network analysis (WGCNA) of the 4143 DEGs identified ten WGCNA network modules. Among them, module2 of 741 member genes showed the highest module-trait (growth habit) association (*r* = 0.86, *p* = 0.0003) in the 12 samples (Figure 6A and Supplementary Table S7). A majority (92.4% or 685) of the 741 member genes comprised DEGs from three groups, including 81 (10.9%) DEGs unique to the comparison between columnar and Std2 (columnar/Std2), 317 (42.7%) DEGs unique to columnar/Std1, and 287 (38.7%) DEGs common to both columnar/Std2 and columnar/Std1 (Figure 6B and Supplementary Table S7). The remaining 56 (7.6%) were from four groups related to Std1. On average, the member genes have 431.9 ± 180.7 edges, ranging from one to 708, in module2. Importantly, the *Co* gene (MD10G1185400) is a member of module2, which is connected by 468 primary neighbor genes (Figure 6C and Supplementary Table S7) that also include five of the seven DEGs under *c2* and four of the five DEGs under *c3* (Figure 5D and Supplementary Table S8), supporting that module2 represents an important gene network responsible for growth habit.

Compared with columnar samples, 639 (86.2%) of the 741 member genes in module2 were downregulated in Std2 while 73 (9.9%) were upregulated and 29 (3.9%) were unchanged (absolute fold change < 1.50). Similarly, 581 (78.4%) of the 741 DEGs were downregulated in Std1, 90 (12.1%) upregulated and 70 (9.4%) unchanged (Supplementary Table S7). These observations indicated that module2 member genes were mostly downregulated in Std1 and Std2 (Supplementary Figure S7), a trend similarly noted in the 588 DEGs between columnar and Std2 (Supplementary Figure S5).

### Enriched MapMan Bins in the *Co* Guided WGCNA Module

Gene enrichment analysis of the member genes in module2 identified 12 MapMan bins that were over-represented (Table 2), which cover 136 of the 741 DEGs (Supplementary Table S7). Among the 12 MapMan bins, M26.9 (misc.glutathione *S*-transferases) and M26.10 (misc.cytochrome P450) are enriched most significantly. Interestingly, five of the 12 DEGs under *c2* and *c3* were found in the 12 MapMan bins. They include MD10G1172300 encoding a glutathione *S*-transferase (GST) TAU 8 like protein in M26.9, and MD09G1174600, MD09G1174700, MD09G1175100, and MD10G1171100 encoding a GDSL lipase in M26.28 (misc.GDSL-motif lipase). Surprisingly, the *Co* gene (MD10G1185400), which controls columnar, is also a member in the enriched M16.8.3 (secondary metabolism.flavonoids.dihydroflavonols). These data suggest that the metabolism of dihydroflavonols and the activities of GSTs, GDSL lipases, and cytochrome P450 proteins likely have a critical role in tree growth habit.

**TABLE 2:** Gene enrichment analyses of WGCNA module2 member genes in MapMan Bins.

MapMan Bins	Description	No. of DEGs expected	No. of DEGs observed	FDR *p*
M26.9	Misc.glutathione *S*-transferases	1	18	1.30E-11
M26.10	Misc.cytochrome P450	6	29	2.22E-09
M30.2.15	Signalling.receptor kinases.thaumatin like	1	13	1.09E-06
M16.8.3	Secondary metabolism.flavonoids.dihydroflavonols	1	11	1.14E-05
M26.28	Misc.GDSL-motif lipase	2	13	7.27E-05
M16.8.2.1	Secondary metabolism.flavonoids.chalcones.naringenin-chalcone synthase	0	5	2.97E-04
M26.6	Misc.*O*-methyl transferases	1	9	0.0019
M26.12	Misc.peroxidases	2	10	0.0076
M21.2	Redox.ascorbate and glutathione	1	8	0.0101
M26.19	Misc.plastocyanin-like	1	7	0.0103
M26.8	Misc.nitrilases, *nitrile lyases, berberine bridge enzymes, reticuline oxidases, troponine reductases	2	9	0.0226
M5.10	Fermentation.aldehyde dehydrogenase	0	4	0.0355
Sum		18	136	

## Discussion

### Mapping of Recessive Traits by Pooled Genome Sequencing in *Malus*

In an effort to adapt the pooled genome sequencing based approach for mapping a dominant trait ‘weeping’ in *Malus*, three segregation types were identified as informative and useful (Dougherty et al., 2018). They include the commonly used <**l**m × mm> (type I) in weeping pool-specific variants, and hidden <**l**m × **ll**> (type II) and <**h**k × **h**k> (type III) in variants common to both weeping and standard pools (Supplementary Table S9). The first allele is designated weeping-linked and the alleles in bold represent a DNA variant in relation to the apple reference genome (Dougherty et al., 2018). However, five segregation types <**h**k × **h**k>(A), <**l**m × **ll**>(B), <**nn** × **n**p>(C), <**l**m × mm>(D), and <pp × **n**p>(E) were inferred as informative for mapping a recessive trait when the approach was adapted in this study (Supplementary Figure S2 and Supplementary Table S3). Since variants under segregation types A-C fall into group Ho-Std2/He-Col and types D-E into group He-Std2/He-Col (Supplementary Table S3), only the variants common to both pools are useful. This implicates that variants specific to pool columnar or Std2 are non-informative, contrasting to the type I variants inferred for dominant traits (Dougherty et al., 2018). Among the five segregation types, type A <**h**k × **h**k> variants are exploited commonly for mapping recessive traits while the types B-E are hidden (Supplementary Figure S2 and Supplementary Tables S3, S9). As to their utilities in mapping recessive traits, segregation types A-C are clearly useful as their variants are exclusively responsible for mapping *c2* and *c3* (Figures 2B,C).

The variants of segregation types <**l**m × mm> (D), and <pp × **n**p> (E) were considered useful. However, their applicability could not be confirmed in this study, suggesting variants of types D and E may not be suitable for mapping recessive traits sometime. Viewing how variants of segregation types B and C were identified, the poor applicability of segregation types D and E might have been caused by the high number of variants (1,636,085) present in group He-Std2/He-Col (Supplementary Table S3) and the filter AF 35–65% used in pool Std2 (Supplementary Table S4). In particular, the filter is of inherent limitations as it targets variants of AF 50%, a variant allele frequency that also could be expected from many variants of unwanted segregation types, such as <**hh** × kk> and <hh × **kk**>. Indeed, the filter AF ≥ 85%, which targets homozygous variants in pool Std2 for segregation types A-C, is much more specific and restrictive, as there were only 70,522 variants in group Ho-Std2/He-Col (Supplementary Table S3). It is recommended that segregation types A-C be the first choices for mapping recessive traits in apple and other species of heterozygous genome.

Mapping the columnar recessive suppressors by mapping their dominant alleles was attempted with two ways to test if recessive model-based approaches were necessary. The first was to use directly the dominant models as described previously (Dougherty et al., 2018). The second was to use variants of segregation types <h**k** × h**k**>, <l**m** × ll>, and <nn × n**p**>, variants of which were inferred as specific to pool columnar (Supplementary Table S3). However, both approaches failed to map *C2* although a 4-Mb region (from the 15th to 19th Mb) overlapping with *c3* on chromosome 9 was detected among several others that could not be confirmed (data not shown). Inspecting the 4-Mb region on chromosome 9 showed that SNVs were markedly reduced in pool Std2 while increased in pool columnar, suggesting that the partial success in mapping of *C3* indeed represent the mapping of *c3*. Nevertheless, this success requires a low degree of heterozygosity in the *c3* region between the reference genome and pool Std2. Given the highly heterozygous nature of the apple genome and the failure to map *c2* using dominance models, it was necessary to use recessive model-based approaches to map recessive traits.

### Homozygous Recessive Loci in Apple, Unique to Crop Plants of Heterozygous Genomes?

This study identified *c2* and *c3* as homozygous genetic loci controlling recessive trait Std2. Despite the high density of homozygous SNVs (AF ≥ 85%) of segregation types A-C, the DNA sequences in the *c2* and *c3* genomic regions in pool Std2 were far from identical. In the *c2* peak region of 2-Mb from the 25th to 27 Mb on chromosome 10, 4710 variants of segregation types A-C were identified (Figures 2B,C), accounting for 52.9% of the total SNVs (8912) common to both pools, i.e., 47.1% were heterozygous SNVs. Similarly, in the *c3* peak region of 2-Mb from the 14th to 16 Mb on chromosome 9, 2160 types A-C variants were identified (Figures 2B,C), accounting for only 46.7% of the total common SNVs (4628). These data suggest that many SNVs remain heterozygous in the *c2* and *c3* regions in pool Std2. This raises the possibility that *c2* and *c3* are recessive compound heterozygous loci as reported commonly in human and animals (Takaku et al., 1998; Zhao et al., 2006; Zhong et al., 2017), which describe a gene locus of two different recessive mutant alleles that confers a recessive condition or disease. However, a high density of homozygous variants of segregation types A-C in the *c2* and *c3* regions were also present in the coding regions of many genes (Supplementary Figures S8, S9). Therefore, it is more likely that the homozygous recessive inheritance of the Std2 trait was determined by the underlying genes carrying homozygous DNA variants. Such recessive loci that are determined by genes of homozygous SNVs in heterozygous genomic regions may reflect an important distinction of apple from the recessive homozygous loci in inbreeding crops, such as rice and tomato, and from the recessive compound heterozygous loci in humans and animals (Takaku et al., 1998; Zhao et al., 2006; Zhong et al., 2017).

### Effect of the *c2* and *c3* Interactions on Columnar Suppression With Incomplete Penetrance

The observed Std2 frequencies in 3 years (2009, 2011, and 2015) were high in double recessive genotype *c2c2 c3c3* (0.867, 0.667, and 0.448), medium in single-recessive carriers *c2c2 c3C3* (0.478, 0.348, and 0.136) and *c2C2 c3c3* and *C2C2 c3c3* (0.237, 0.186, and 0.153), and low in non-recessive carriers *c2C2 c3C3* and *C2C2 c3C3* (0.107, 0.089, and 0.018) (Figure 4). Since the frequency of Std2 in the double recessive carrier was even more than the combined fractions of *c2c2* and *c3c3* in single recessive carriers in the 3 years (0.715, 0.534, and 0.289), the two loci were proposed to suppress columnar through additive gene interactions. The hypothesis is that the homozygous recessive genotypes *c2c2* and *c3c3* each would drive a certain fraction of the single recessive carriers to express Std2 at a given year while the double recessive genotype *c2c2 c3c3* would empower a higher fraction or all of its carriers to express Std2. Overall, this proposal explains the data well although the small fraction of Std2 in non-recessive carries could not be accounted for. Apparently, the Std2 frequencies in the double recessive carrier *c2c2 c3c3* were lower than 100% in the 3 years, suggesting the additive effect of *c2* and *c3* could drive only “incomplete penetrance” of Std2 that could be reduced to a lower penetrance by tree age.

Incomplete penetrance and variable expressivity have been documented well in plants (Sekhon and Chopra, 2009; Mazzucato et al., 2015), animals (Eichers et al., 2006; Raj et al., 2010), and humans (Bourgeois et al., 1998; Giudicessi and Ackerman, 2013). Depending upon studies, the range of incomplete penetrance varied widely. For example, the range of penetrance for human long QT syndrome (LQTS) in individual LQTS families were between 25% and 100% (Giudicessi and Ackerman, 2013), whereas the penetrance of aberrations in cotyledon morphology and carpelloid stamens in homozygous siblings (BC_1_F_2_) from an *Aux/IAA9* frameshift mutation in tomato were 47.1 and 41.0%, respectively (Mazzucato et al., 2015). In addition, age-dependent penetrance and expressivity of certain phenotype appeared to be common in animals (Eichers et al., 2006) and plants (Ashri, 1970) as well. The causal factors for the phenomenon of incomplete penetrance have been attributed to environments, interactions with other genes, and epigenetic regulation of expression of the underlying genes (Lalucque and Silar, 2004; Raj et al., 2010; Wittmeyer et al., 2018). Since the retroposon induced expression of the *Co* gene in columnar (MD10G1185400) (Wolters et al., 2013; Otto et al., 2014; Okada et al., 2016) was drastically suppressed in Std2 (Figure 5E and Supplementary Figure S5A), it is possible that *c2* and *c3* would interact with *Co* and/or involve an epigenetic mechanism that regulates the expression of *Co*, thereby the penetrance of phenotype Std2.

### Candidate Genes Under *c2* and *c3*

The DEGs between columnar and Std2 under *c2* and *c3* (Figure 5D) are considered an important group of candidate genes, of which the four GDSL lipase encoding genes (MD09G1174600, MD09G1174700, MD09G1175100, and MD10G1171100) are of particular interest as they are also members in the enriched MapMan bin M26.9 (Table 2 and Supplementary Table S7). Under *c3*, the three GDSLs were all downregulated in Std1 and Std2 and were expressed at relatively lower levels, similar to the *Co* gene (Figures 5D,E). The *Arabidopsis* counterpart of MD09G1174600 is At1g53940 (GLIP2, AtGELP20), and that of both MD09G1174700 and MD09G1175100 is At5g40990 (GLIP1, AtGELP97). Interestingly, GLIP1 and GLIP2 are most closely related member genes of Clade IIIa in the GDSL lipase gene family in *Arabidopsis* (Lai et al., 2017), suggesting the three GDSLs form a closely related gene cluster under *c3*. T-DNA knockout lines of At1g53940 (GLIP2) and At5g40990 (GLIP1) were similarly more sensitive to pathogen *E. carotovora* than their wild type controls (Lee et al., 2009; Lai et al., 2017), implicating their roles in plant response to biotic stress. However, the T-DNA knockout lines of At1g53940 (GLIP2) were observed also with drastically increased lateral roots, impaired gravitropic response of shoots, and increased levels of the transcripts of *IAA1* and *IAA2*, indicating At1g53940 (GLIP2) negatively regulates auxin signaling (Lee et al., 2009), which is important in plant growth and development.

MD10G1171100 under *c2* showed an opposite expression profile of the three GDSLs under *c3*. The *Arabidopsis* counterpart of MD10G1171100 is At4G28780 (AtGELP82), which is a member in Clade IIb of the GDSL lipase gene family (Lai et al., 2017). This clade includes a well-characterized gene, At5g33370 (AtGELP95) that encodes CUTIN SYNTHASE2 (CUS2), which is essential for the development of cuticular ridges in *Arabidopsis* sepals (Hong et al., 2017). *CUS2* is mostly expressed in various organs in reproductive stage while At4G28780 is expressed in many tissues in both vegetative and reproductive stages (Schmid et al., 2005), implicating a complex role of the Clade IIb genes in plant development.

In addition, non-DEGs MD10G1165100 and MD09G1170200 are of interest as they are transcription regulators with at least one non-synonymous mutation (Supplementary Table S8). This is particular true considering the repressed *Co* gene expression in Std2 and the incomplete penetrance of the Std2 phenotype. MD10G1165100 encodes a LEUNIG_Homolog (LUH)-like transcriptional corepressor closely related to LEUNIG (LUG) (At4g32551) and LEUNIG_HOMOLOG (LUH) (At2g32700) in *Arabidopsis*. LEUNIG (LUG) (At4g32551) represses AGAMOUS expression during flower development by forming a LEUNIG-SEUSS repression complex in Arabidopsis (Liu and Meyerowitz, 1995; Conner and Liu, 2000; Sridhar et al., 2004). More importantly, LEUNIG-SEUSS repression complex also includes LEUNIG_HOMOLOG (LUH) (At2g32700) and SEUSS-like proteins and physically interacts with transcription factor YABBYs (Stahle et al., 2009), which are crucial in regulation of plant shoot apical meristem partitioning and organization and lateral organ development (Goldshmidt et al., 2008; Bonaccorso et al., 2012; Tanaka et al., 2012).

MD09G1170200 is an apple ortholog of AT1G79020 encoding EPL1B, a subunit evidently in the NuA4 (nucleosome acetyltransferase of H4) histone acetyltransferase complex (Bieluszewski et al., 2015). The NuA4 complex, which includes six essential subunits: Esa1, Epl1, Tra1, Arp4, Act1, and Swc4, are broadly conserved in eukaryotes, is responsible for acetylation of histone H4 and H2A N-terminal tails (Doyon et al., 2004; Searle et al., 2017). The orthologs of the yeast Epl1 subunit include EPC1 in human and E(Pc) (enhancer protein of polycomb) in *Drosophila melanogaster*. A study in yeast uncovered that the interactions between subunits Epl1 and Esa1 are essential for chromatin regulation (Searle et al., 2017). As discussed above, epigenetic regulation has been an important mechanism responsible for incomplete penetrance. It is possible that MD09G1170200 would be involved in epigenetic regulation of the *Co* gene expression in individuals that are single or double recessive carriers of *c2* and *c3*. Nevertheless, more dedicated studies are needed to determine if any of the candidate genes discussed are the casual genes underlying *c2* and *c3* that suppress the *Co* gene expression and columnar phenotype.

## Conclusion

By exploring DNA variant segregation types in pooled genome sequencing, this study elucidated the genetic basis on which SNVs of segregation types A-E can be employed together with the AFDDD mapping strategy to map recessive traits in apple. Application of the mapping strategy successfully identified two recessive suppressors (genes) *c2* and *c3* associated with columnar suppression, which are located on chromosomes 10 and 9, respectively. An important mechanism through which *c2* and *c3* mediate the columnar suppression is to suppress the *Co* gene expression. The identification of the *Co* gene-guided WGCNA module offers further clues on how the causal genes underlying *c2* and *c3* may function to repress the *Co* gene expression. Overall, this study demonstrates an effective approach for mapping recessive traits in apple and other out-crossing crop species and provides new insights into genetic and molecular regulation of columnar growth habit in apple.

## Data Availability Statement

The datasets presented in this study can be found in online repositories. The names of the repository/repositories and accession number(s) can be found: NCBI Project accession: PRJNA547442.

## Author Contributions

KX conceived the study. LD and TB conducted the experiments. LD, TB, and KX analyzed the data and wrote the manuscript. SB created the mapping populations. All authors read and approved the final manuscript.

## Conflict of Interest

The authors declare that the research was conducted in the absence of any commercial or financial relationships that could be construed as a potential conflict of interest.

## Abbreviations

AFDD: allele frequency directional difference
AFDDD: AFDD and density
DEGs: differentially expressed genes
SNVs: single nucleotide variants
WGCNA: weighted gene co-expression network analysis.
