# Supplementary material for: Exploring DNA Variant Segregation Types Enables Mapping Loci for Recessive Phenotypic Suppression of Columnar Growth in Apple
Source: Front Plant Sci. 2020 Jun 9;11:692. doi: 10.3389/fpls.2020.00692 (PMC7297030; doi:10.3389/fpls.2020.00692)
Supplement: Supplementary file 1 [file Data_Sheet_1.pdf]

Figure S1

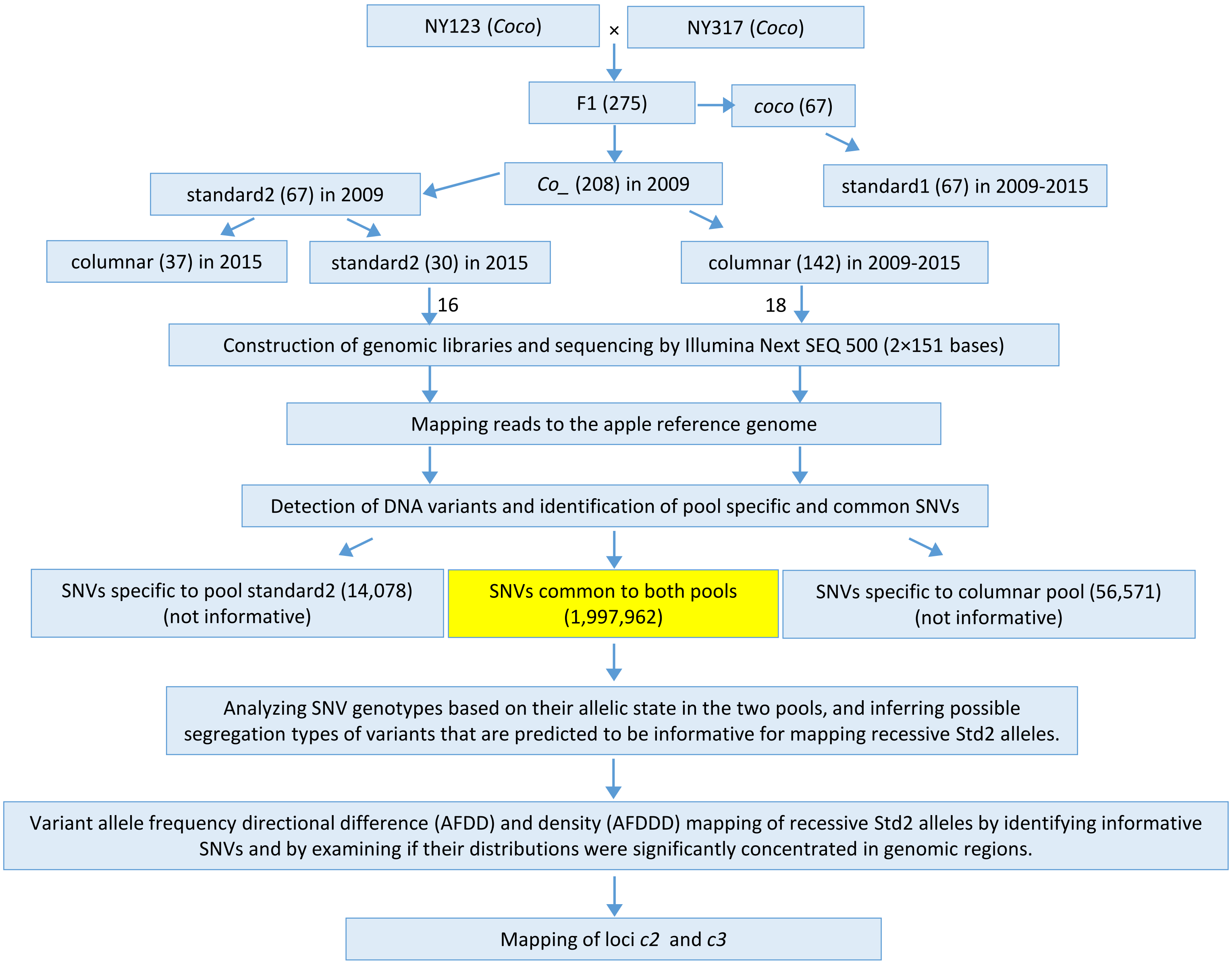

Figure S1. A flowchart illustrating the procedure in pooled genome sequencing and variant allele frequency directional difference (AFDD) and density (AFDDD) mapping of the recessive standard2 (Std2) phenotype. Single nucleotide variants (SNVs) were identified with the following settings: reads overage =20-200; no complex genotype and variant allele frequency (AF)≥15%. Pool specific variants were filtered further against the reads mappings in the contrasting pool in which variant AF≤10% was set.

Figure S2

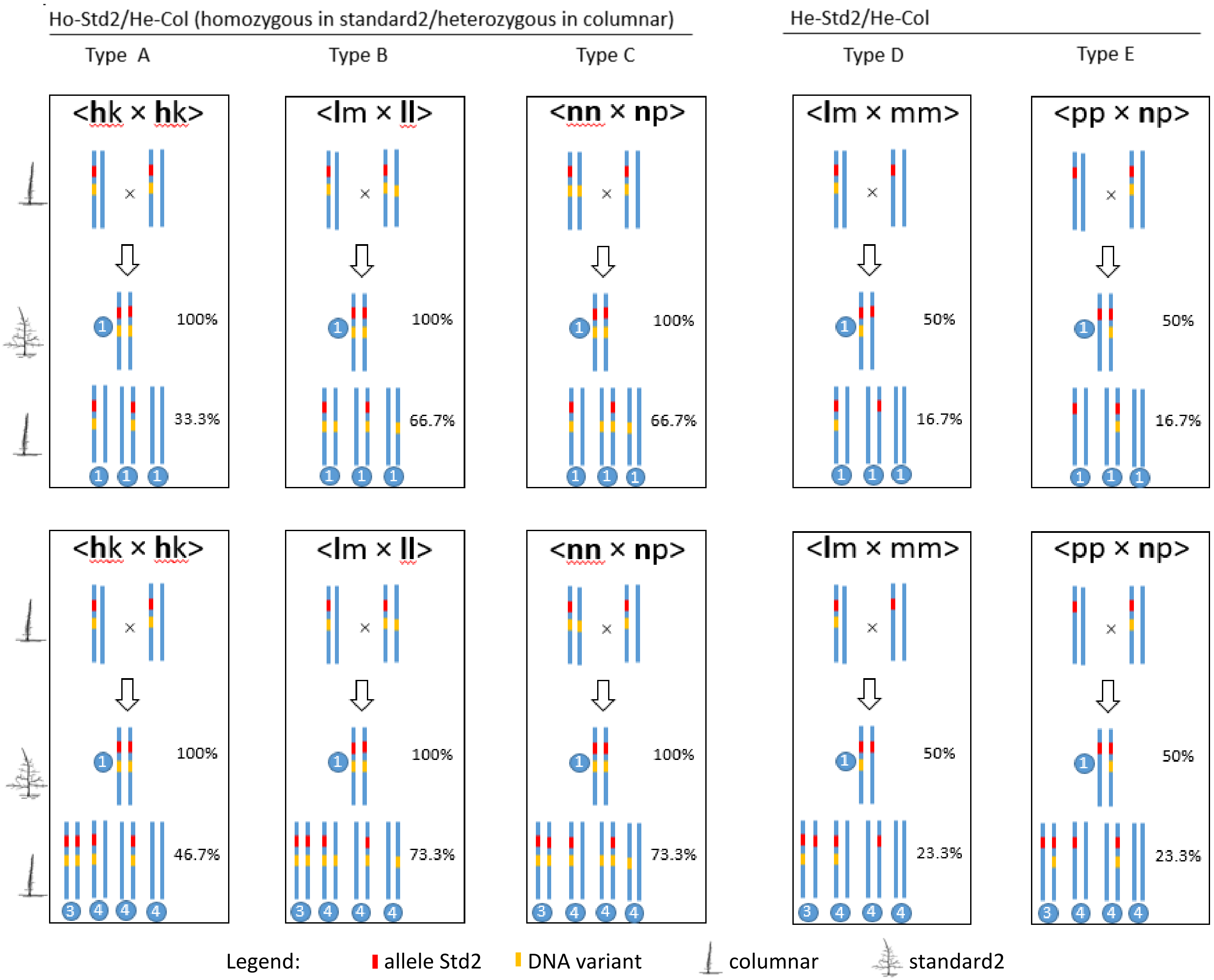

Figure S2. Schematic representations of informative variant segregation types (types A-E) inferred from SNV groups Ho-Std2/He-Col (homozygous in pool standard2/heterozygous in pool columnar) and He-Std2/He-Col under models of one (top panel) and two (bottom panel) recessive repressors. Non-informative segregation types were listed in Supplementary Table S3. Each segregation type is illustrated in a rectangle box that includes the two parents at the top, one representative standard2 progeny in the middle, and three or four columnar progenies at the bottom. The long vertical lines in blue represent the chromosomal segment harboring the recessive repressor locus. The red and orange short vertical lines represent the recessive repressor allele(s) and DNA variants in relation to the reference genome, respectively. The tree-drawings indicate columnar and standard2 phenotypes, respectively. The numbers within the blue circles stand for the genotype fraction number in the progeny. Total fractions under one- and two-gene model are 4 and 16, respectively. The expected allele frequencies (%) of DNA variants in pools standard2 and columnar are given accordingly. In segregation type denotation, each letter denotes one of the four DNA bases and the alleles in each first and third positions are assumed in linkage with the recessive standard2 alleles in the seed and pollen parents, respectively. Std2: standard2; Col: columnar.

Figure S3

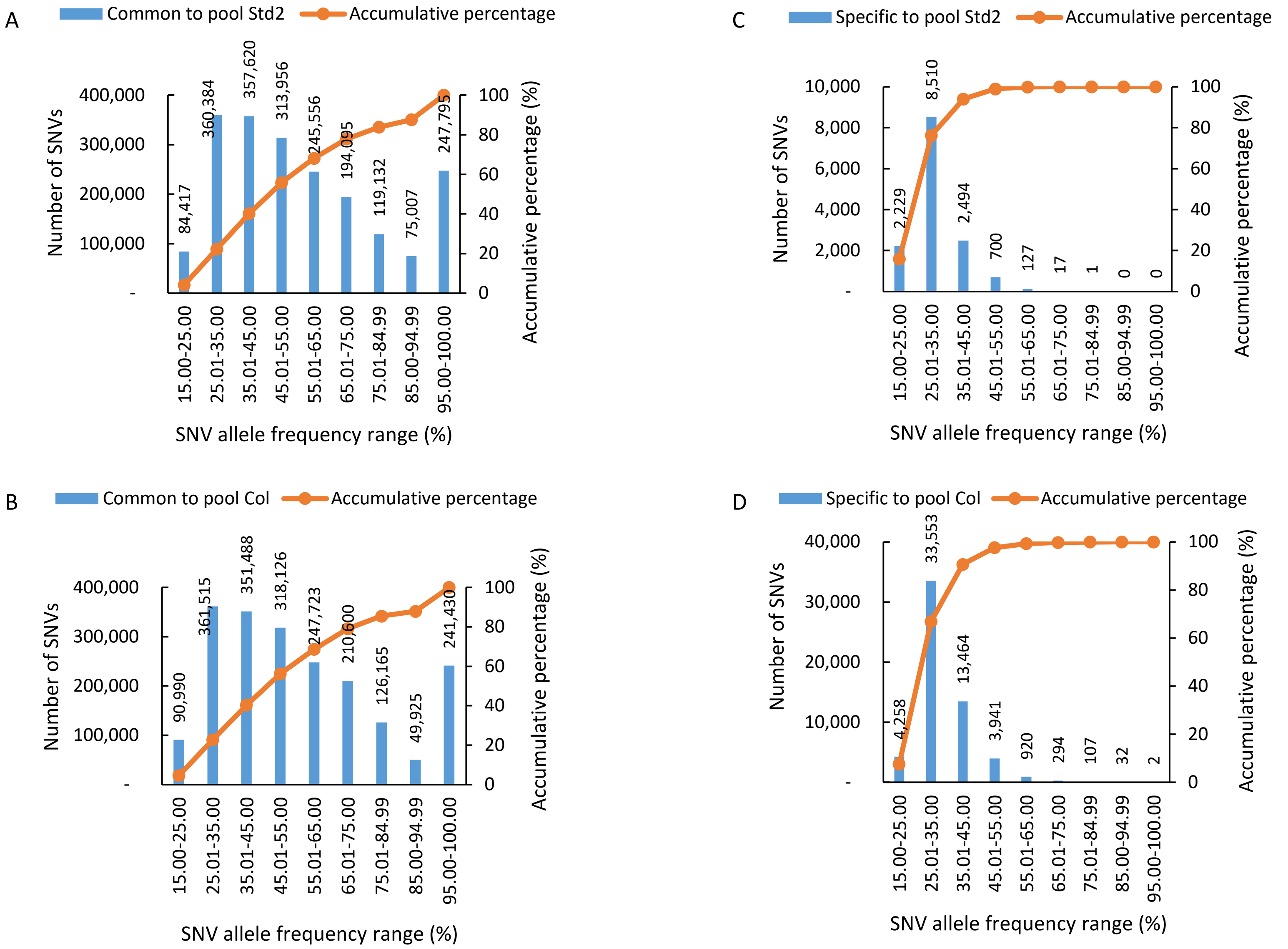

Figure S3. Distribution of single nucleotide variants (SNVs) under various SNV allele frequencies. (A and B) Distribution of the 1,997,962 SNVs common to both pools in pool Std2 (A) and columnar (B). (C and D) Distribution of pool specific SNVs in pool Std2 (14,078 SNVs) (C) and pool columnar (56,571 SNVs) (D).

Figure S4

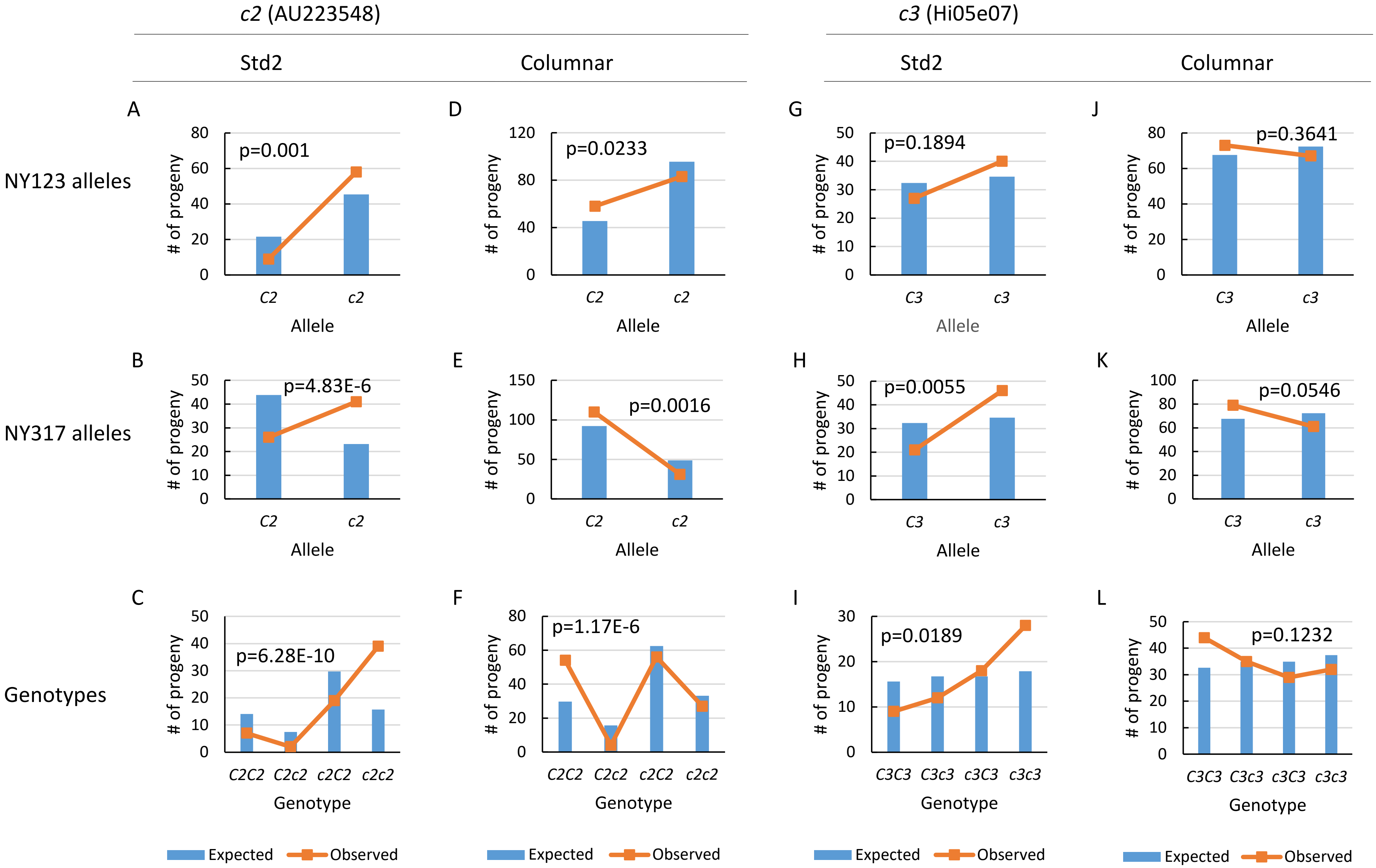

Figure S4. The expected and observed frequencies of alleles *c2* and *c3* and their genotypes in the standard2 (Std2) and columnar sub-populations in 2009. (A-F) The expected and observed frequencies of alleles *c2* from parents NY123 (A, D) and NY317 (B, E) in sub-populations Std2 (A, B) and columnar (D, E), and the expected and observed *c2* genotype frequencies in sub-populations Std2 (C) and columnar (F). (G-L) The expected and observed frequencies of alleles *c3* from parents NY123 (G, J) and NY317 (H, K) in sub-populations Std2 (G, H) and columnar (J, K), and the expected and observed *c3* genotype frequencies in sub-populations Std2 (I) and columnar (L). The p values indicate levels of significance in chi-square test. The *c2* and *c3* loci were represented and investigated with markers AU223548 and Hi05e07, respectively.

Figure S5

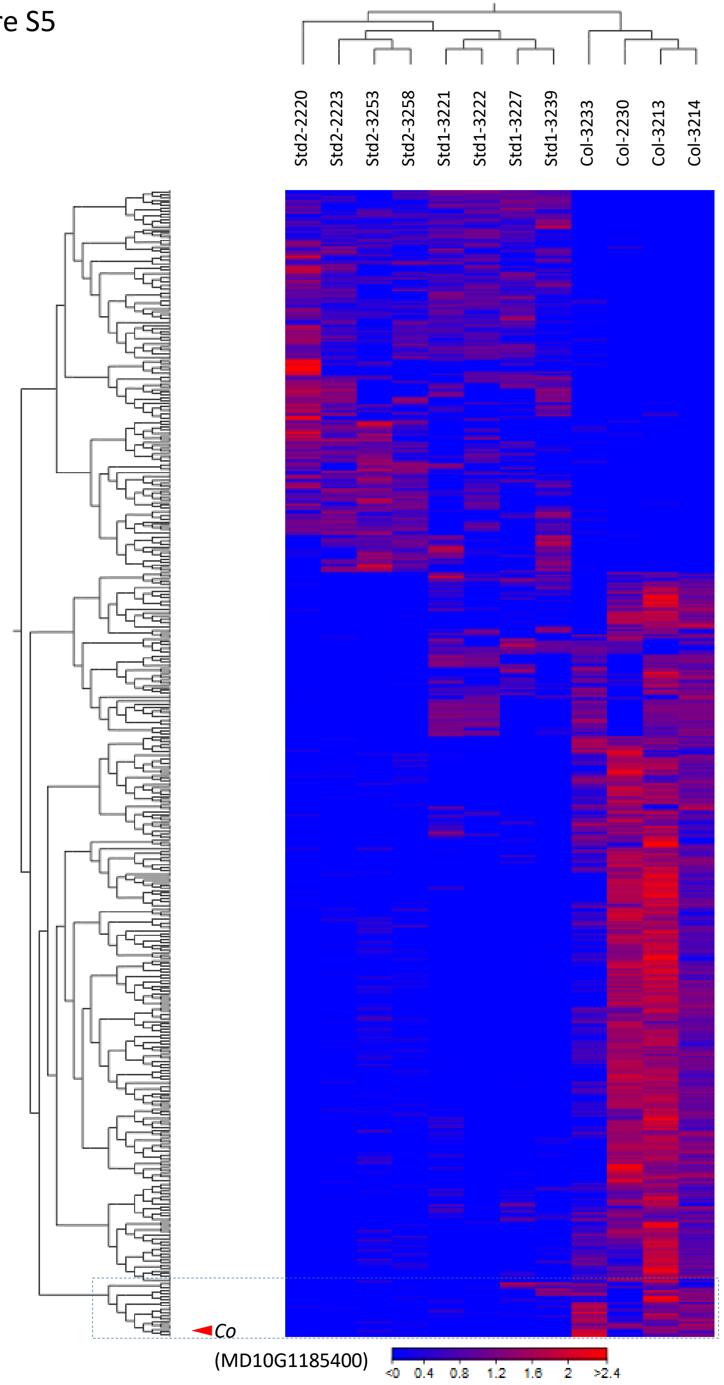

Figure S5. Heat map representation of DEGs (588) between columnar and standard2 progeny. There are 196 DEGs up-regulated and 392 down-regulated in Std2, respectively. The hierarchical cluster trees indicate the relationships among genes (left) and samples (top), respectively. The broken-line boxes show the section containing the *Co* gene (indicated by a red arrow) in the overall and zoom-in views, respectively. The arrows in blue indicate DEGs in the *c3* region.

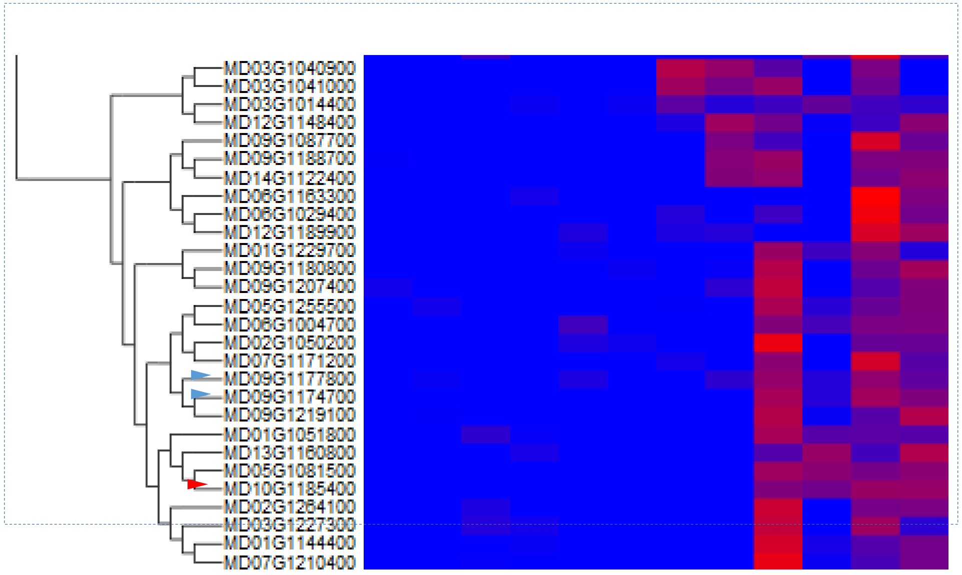

Figure S6

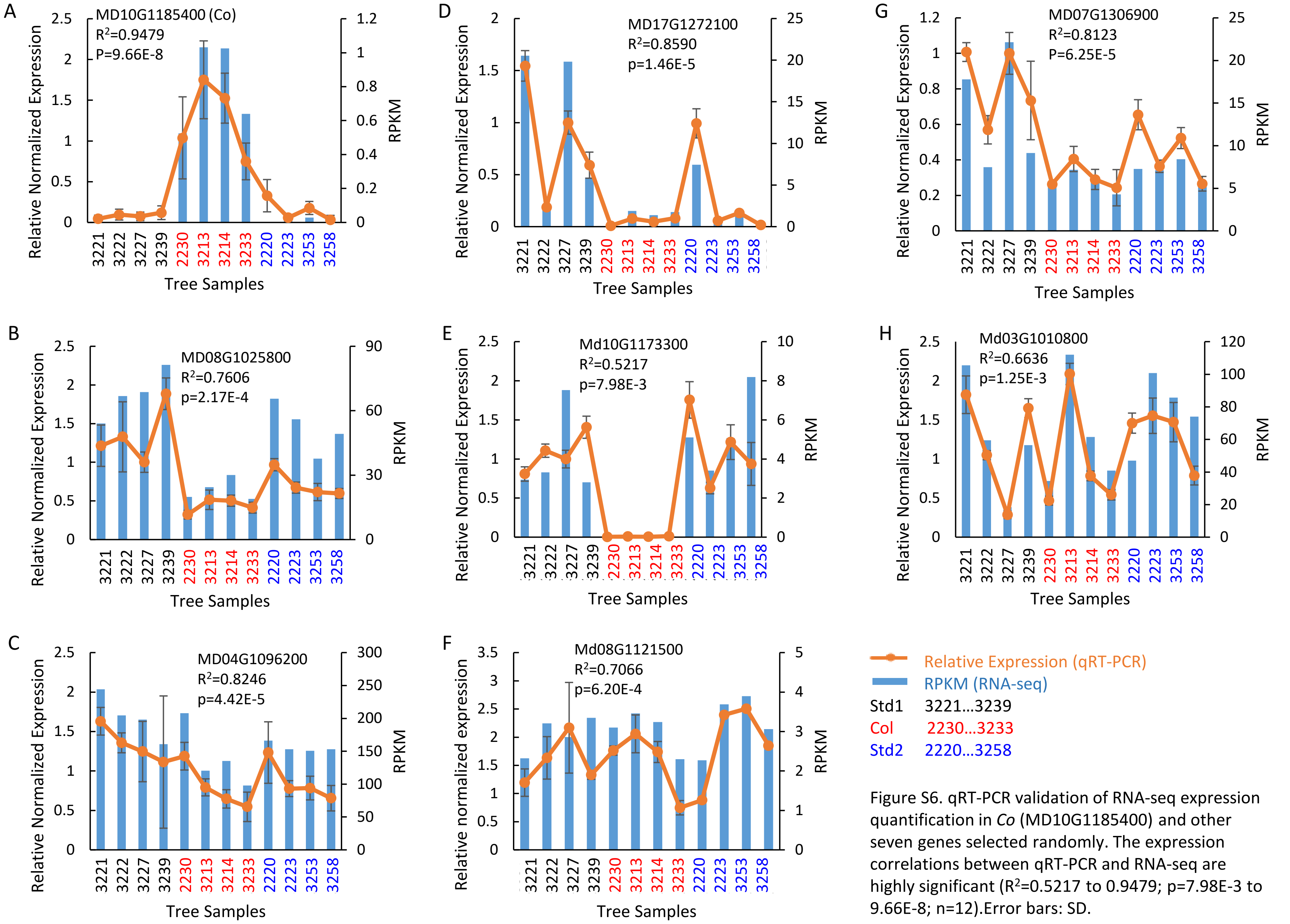

Figure S6. qRT-PCR validation of RNA-seq expression quantification in *Co* (MD10G1185400) and other seven genes selected randomly. The expression correlations between qRT-PCR and RNA-seq are highly significant (R^2=0.5217 to 0.9479; p=7.98E-3 to 9.66E-8; n=12).Error bars: SD.

Figure S7

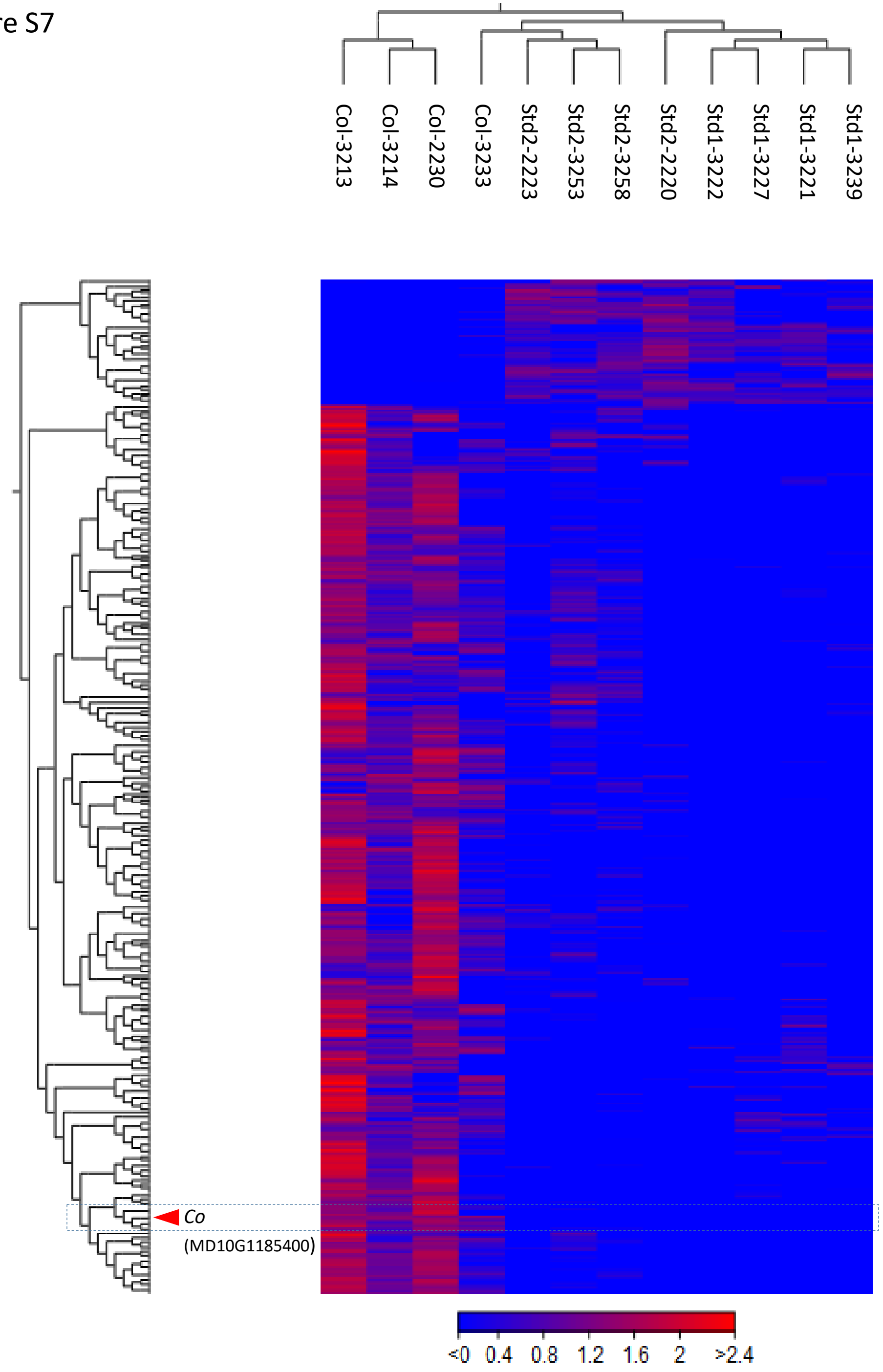

Figure S7. Heat map representation of DEGs (741) in WGCNA module2 in which the Co gene is a member. 639 (86.2%) of the 741 member genes in module2 were downregulated in Std2 while 73 (9.9%) were upregulated and 29 (3.9%) were unchanged (fold change <1.50). The hierarchical cluster trees indicate the relationships among genes (left) and samples (top), respectively. The broken-line boxes show the section containing the *Co* gene (indicated by a red arrow) in the overall and zoom-in views, respectively.

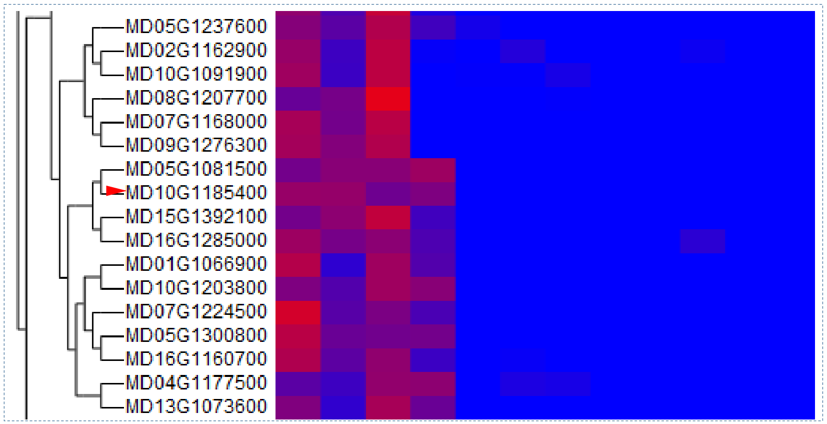

Figure S8

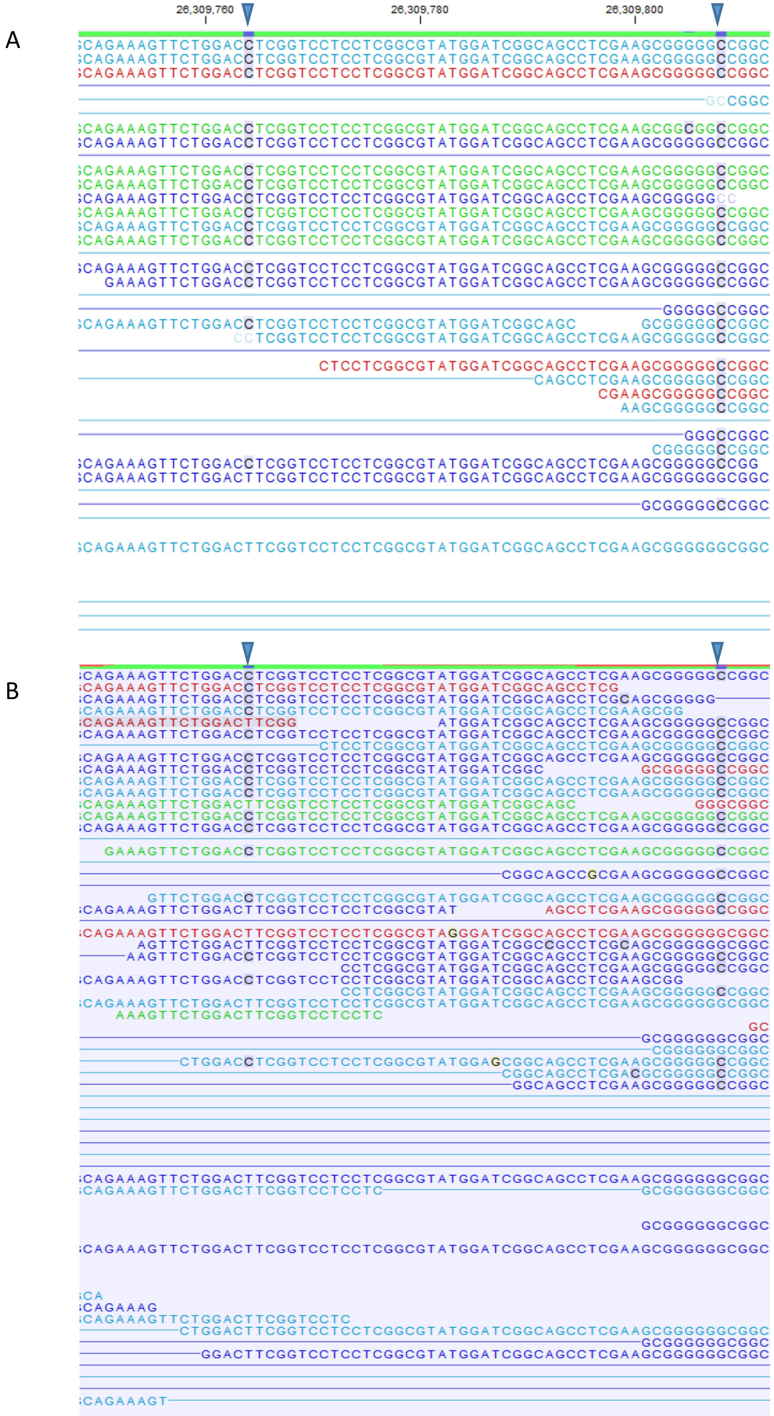

Figure S8. A screen snapshot of reads mapping in pools Std2 (A) and columnar (B) in a coding region of gene MD10G1170600 (AU223548) at locus *c2*. Note that the two SNVs (indicated by arrows) are homozygotes in pool Std (A) while heterozygous in pool columnar (B).

Figure S9

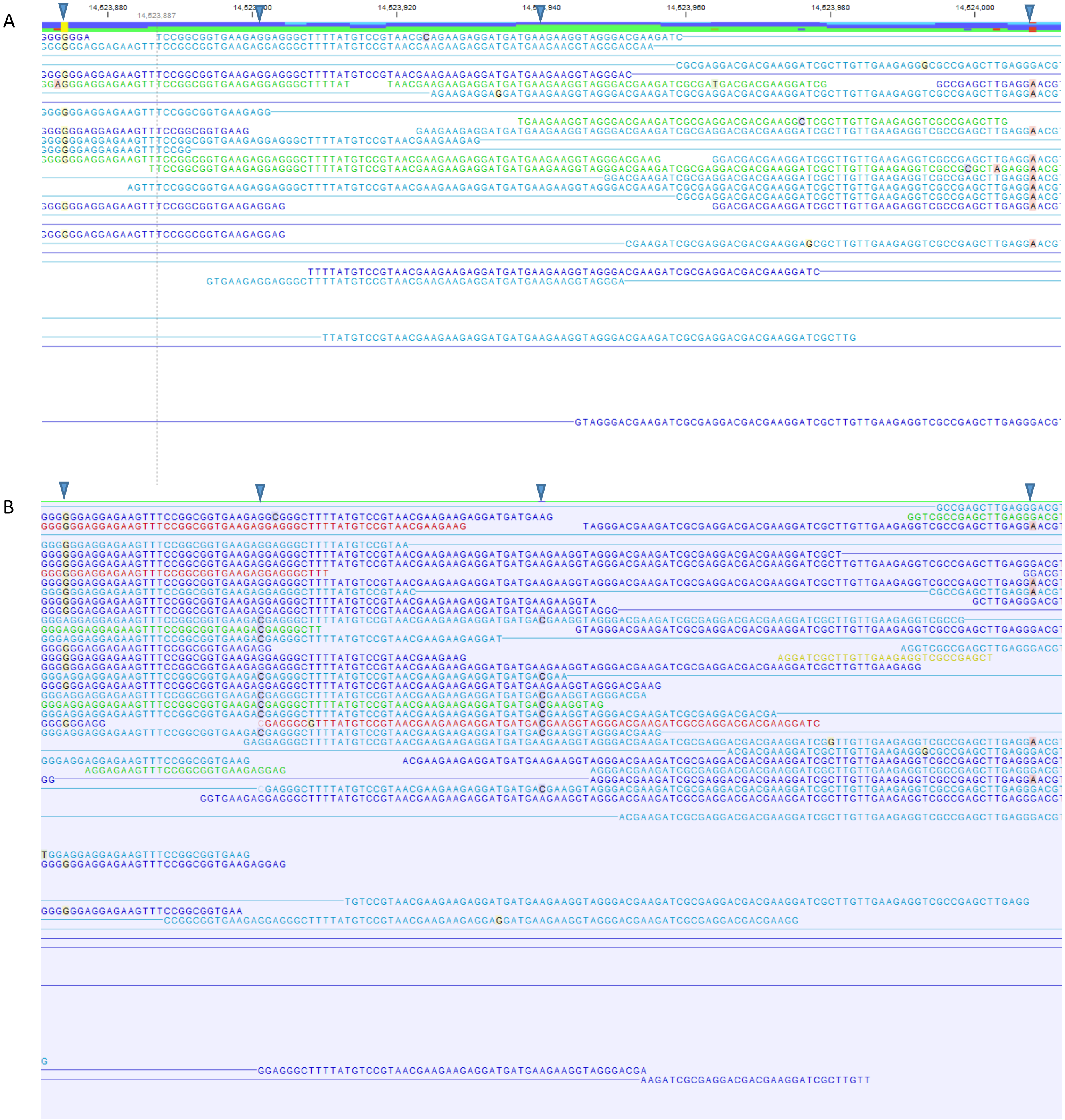

Figure S9. A screen snapshot of reads mapping in pools Std2 (A) and columnar (B) in a coding region of gene MD09G1171900 at locus *c3*. Note that the two SNVs and two non-SNVs (indicated by arrows) are homozygotes in pool Std (A) while heterozygous in pool columnar (B).

Table S1. F1 progeny used in pooled genome sequencing and their genotypes at the *Co* locus and growth habit.

Progeny #	*Co* Genotype	Growth habit-2009 (2-year-old)	Growth habit-2011 (4-year-old)	Growth habit-2015 (8-year-old)	Pool
2212	***CoCo***	C	C	C	Columnar
2237	***CoCo***	C	C	C	Columnar
2265	*Coco*	C	C	C	Columnar
2241	*Coco*	C	C	C	Columnar
2213	*Coco*	C	C	C	Columnar
1162	*Coco*	C	C	C	Columnar
2248	*Coco*	C	C	C	Columnar
2214	*Coco*	C	C	C	Columnar
2233	*Coco*	C	C	C	Columnar
2238	*Coco*	C	C	C	Columnar
2240	*Coco*	C	C	C	Columnar
2243	*Coco*	C	C	C	Columnar
2244	*Coco*	C	C	C	Columnar
2264	*Coco*	C	C	C	Columnar
2266	*Coco*	C	C	C	Columnar
2229	*Coco*	C	C	C	Columnar
1178	*Coco*	C	C	C	Columnar
1179	*Coco*	C	C	C	Columnar
2279	*Coco*	S	S	SL	Std2
2290	*Coco*	S	S	SL	Std2
2298	*Coco*	S	S	SL	Std2
2304	*Coco*	S	S	SL	Std2
2253	*Coco*	S	S	S	Std2
2252	*Coco*	S	S	S	Std2
2258	*Coco*	S	S	S	Std2
2269	*Coco*	S	S	S	Std2
2281	*Coco*	S	S	S	Std2
2287	*Coco*	S	S	S	Std2
2316	*Coco*	S	S	S	Std2
1159	*Coco*	S	S	S	Std2
1131	*Coco*	S	S	S	Std2
2313	*Coco*	S	S	S	Std2
2273	***CoCo***	S	S	S	Std2
2321	***CoCo***	S	S	S	Std2

C: columnar; S: standard; SL: standard like. Std2: Standard2

Table S2. Illumina raw and clean reads obtained, and statistics of read mapping against the apple reference genome

Pool		Count	Percentage of reads	Average length	Number of bases	Percentage of bases	Mean read length (bases)	seq. depth (×)
Standard2	Raw reads	166,523,370			25,145,028,870		151	35.4
	Clean reads	163,545,102	100.00%	124.95	20,435,067,649	100.00%	125.0	28.8
	**Mapped reads**	122,944,524	75.17%	129.63	15,937,142,369	77.99%		22.5
	Not mapped reads	40,600,578	24.83%	110.78	4,497,925,280	22.01%		
	Reads in pairs	90,133,668	55.11%	446.75	12,095,345,261	59.19%		
	Broken paired reads	32,810,856	20.06%	117.09	3,841,797,108	18.80%		
columnar	Raw reads	259,133,758			39,129,197,458		151	55.1
	Clean reads	253,038,996	100.00%	125.8	31,833,442,891	100.00%	125.8	44.9
	**Mapped reads**	187,827,369	74.23%	130.6	24,529,375,576	77.06%		34.6
	Not mapped reads	65,211,627	25.77%	112.01	7,304,067,315	22.94%		
	Reads in pairs	138,048,096	54.56%	402.77	18,736,289,830	58.86%		
	Broken paired reads	49,779,273	19.67%	116.38	5,793,085,746	18.20%		

Reference genome size: 709,561,391 bp

Table S3. Genotypes of variants common to both pools and variant segregation type inferred (with heterozygous parents)

Variant genotype group	Variant genotypes observed[a]				Inferred[b]								Notes
	Std2 pool	Col pool	# of variants (1,997,962 in total)	% of variants (freq.)	Segregation types (genotype of parents)[c]	Std2 pool genotype	Std2 pool mean AF (%)	Col pool genotype			Col pool mean AF (%)	AFDD between Std2 and col pools (perecentage points)	
one recessive gene													
Ho-Std2/He-Col	Ho-Std2	He-Col	70,522	3.53	<**h**k x **h**k>	**hh**	100	2**h**k	kk		33.3	66.7	Informative for Std2 (in variants common to both pools)
					<h**k** x h**k**>	hh	0	2h**k**	**kk**		66.7	-66.7	Informative for Col (in variants specific to pool columnar)
					<**l**m x **ll**>	**ll**	100	**ll**	m**l**	m**l**	66.7	33.3	Informative for Std2 (in variants common to both pools)
					<l**m** x ll>	ll	0	ll	**m**l	**m**l	33.3	-33.3	Informative for Col (in variants specific to pool columnar)
					<**nn** x **n**p>	**nn**	100	**nn**	**n**p	**n**p	66.7	33.3	Informative for Std2 (in variants common to both pools)
					<nn x n**p**>	nn	0	nn	np	n**p**	33.3	-33.3	Informative for Col (in variants specific to pool columnar)
He-Std2/He-Col	He-Std2	He-Col	1,636,085	81.89	<**hh** x kk>	**h**k	50	2**h**k	**h**k		50	0	Not informative (in variants common to both pools)
					<hh x **kk**>	h**k**	50	2h**k**	h**k**		50	0	Not informative (in variants common to both pools)
					<**l**m x mm>	**l**m	50	**l**m	mm	mm	16.7	33.3	Informative for Std2 (in variants common to both pools)
					<l**m** x **mm**>	l**m**	50	l**m**	**mm**	**mm**	83.3	-33.3	Informative for Col (in variants common to both pools)
					<pp x **n**p>	**n**p	50	pp	pp	**n**p	16.7	33.3	Informative for Std2 (in variants common to both pools)
					<**pp** x n**p**>	n**p**	50	**pp**	**pp**	n**p**	83.3	-33.3	Informative for Col (in variants common to both pools)
He-Std2/Ho-Col[e]	He-Std2	Ho-Col	39,075	1.96	NA								
Ho-Std2/Ho-Col	Ho-Std2	Ho-Col	252,280	12.63	<**qq** x **qq**>	**qq**	100	**qq**	**qq**	**qq**	100	0	Not informative (in variants common to both pools)
Two recessive genes													
Ho-Std2/He-Col	Ho-Std2	He-Col	70,522	3.53	<**h**k x **h**k>	**hh**	100	8**h**k	4kk	3**hh**	46.7	53.3	Informative for Std2 (in variants common to both pools)
					<h**k** x h**k**>	hh	0	8h**k**	4**kk**	3hh	53.3	-53.3	Informative for Col (in variants specific to pool columnar)
					<**l**m x **ll**>	**ll**	100	7**ll**	4m**l**	4m**l**	73.3	26.7	Informative for Std2 (in variants common to both pools)
					<l**m** x ll>	ll	0	7ll	4**m**l	4**m**l	26.7	-26.7	Informative for Col (in variants specific to pool columnar)
					<**nn** x **n**p>	**nn**	100	7**nn**	4**n**p	4**n**p	73.3	26.7	Informative for Std2 (in variants common to both pools)
					<nn x n**p**>	nn	0	7nn	4n**p**	4n**p**	26.7	-26.7	Informative for Col (in variants specific to pool columnar)
He-Std2/He-Col	He-Std2	He-Col	1,636,085	81.89	<**hh** x kk>	**h**k	50	8**h**k	4**h**k	3**h**k	50	0	Not informative (in variants common to both pools)
					<hh x **kk**>	h**k**	50	7h**k**	4h**k**	4h**k**	50	0	Not informative (in variants common to both pools)
					<**l**m x mm>	**l**m	50	7**l**m	4mm	4mm	23.3	26.7	Informative for Std2 (in variants common to both pools)
					<l**m** x **mm**>	l**m**	50	7l**m**	4**mm**	4**mm**	76.7	-26.7	Informative for Col (in variants common to both pools)
					<pp x **n**p>	**n**p	50	8pp	4**n**p	3**n**p	23.3	26.7	Informative for Std2 (in variants common to both pools)
					<**pp** x n**p**>	n**p**	50	8**pp**	4n**p**	3n**p**	76.7	-26.7	Informative for Col (in variants common to both pools)
He-Std2/Ho-Col	He-Std2	Ho-Col	39,075	1.96	NA								
Ho-Std2/Ho-Col[d]	Ho-Std2	Ho-Col	252,280	12.63	<**qq** x **qq**>	**qq**	100	8**qq**	4**qq**	3**qq**	100	0	Not informative (in variants common to both pools)

[a] Homozygous (Ho): variant allele frequency (AF)>85%; Heterozygous (He): 15%≤ AF≤85%

[b] for variants in the genomic regions responsible for phenotype standard2.

[c] The alleles in each first and third positions are assumed in linkage with the recessive Std2 alleles (repressors of columnar) in the seed and pollen parents, respectively, and those in bold are a polymorphic variant in relation to the apple reference genome. Complex segregation types <ab × cd> and <ee × fg> involving simultaneously three or four DNA bases are not considered due to their relative low frequency in the genome. Based on allele frequency directional difference (AFDD) inferred, five segregation types <**h**k × **h**k> (A), <**l**m × **ll**> (B), <**nn** × **n**p> (C), <**l**m × mm> (D), and <pp × **n**p> (E) were considered informative for mapping the recessive traits in apple under the model of one- or two-recessive genes. Filtering informative variants was detailed in Table S4.

[d] The existence of such variant genotype group was considered unlikely. The variants observed were likely due to the leak-through of other segregation types, such as <l**m** × **mm**> and <**pp** × n**p**> in the variant genotype group He-Std2/He-Col, which are expected to have high variant allele frequencies 83.3% and 73.3% (close to the 85% threshold for homozygotes) under the model of one- and two-recessive genes, respectively.

Std2: standard2; Col: columnar; AFDD: allele frequency directional difference.

Table S4. Filters used for identification of informative variants

Segregation types	Symbol	No. of recessive genes (model)	Std2 pool mean AF (%)			Col pool mean AF (%)	AFDD between Std2 and columnar pools (percentage points)			No. of variants identified
			Expected	Targeted	Used	Expected	Expected	Targeted	Used	
A	<hk × hk>	1	100	≥85	≥85	33.3	66.7	56.7 to 76.7	≥43.3	7,642
		2	100	≥85		46.7	53.3	43.3 to 63.3		
B	<lm × ll>	1	100	≥85	≥85	66.7	33.3	23.3 to 43.3	16.7 to 43.3	40,166
		2	100	≥85		73.3	26.7	16.7 to 36.7		
C	<nn × np>	1	100	≥85		66.7	33.3	23.3 to 43.3		
		2	100	≥85		73.3	26.7	16.7 to 36.7		
D	<lm × mm>	1	50	35 to 65	35 to 65	16.7	33.3	23.3 to 43.3	16.7 to 43.3	70,230
		2	50	35 to 65		23.3	26.7	16.7 to 36.7		
E	<pp × np>	1	50	35 to 65		16.7	33.3	23.3 to 43.3		
		2	50	35 to 65		23.3	26.7	16.7 to 36.7		
Sum										118,038

For identification of DNA variants under segregation type A, the cut-off is AFDD ≥43.3, ten percentage points lower than AFDD 53.3 to accommodate variations, which is estimated under model of two recessive genes. Consequently, 7,642 informative SNVs were identified among the 70,522 variants in genotype group Ho-Std2/He-Col (Table S3). For DNA variants under segregation types B-C, the cut-off is AFDD 16.7, ten percentage points lower than AFDD 26.7 to accommodate variations, which is estimated under model of two recessive genes. This led to identification of 40,166 SNVs in variant genotype group Ho-Std2/He-Col (Table S3). For segregation types D-E, 70,230 of the 1,636,085 SNVs in variant genotype group He-Std2/He-Col (Table S3) were identified. These SNVs were obtained using the following filters: 1) the variant AF range is from 35% to 65% in pool standard2, close to their estimated mean 50%. 2) The AF is no higher than 33.3% in pool columnar, ten percentage points higher than 23.3% estimated for two recessive genes. 3) The cut-off is AFDD 16.7 between pools standard2 and columnar.

Table S5. List of primers

Name of markers or target genes	Forward Primer (5' to 3')	Reverse Primer (5' to 3')	Genome location	Purpose
CH02c11	TGAAGGCAATCACTCTGTGC	TTCCGAGAATCCTCTTCGAC	Ch10: 24,261 kb	Confirmation of c2-SSR
Ch10_24818	ACCAAACCAAGACACATGCT	GGGGTTATTTACTGTGGTGGTG	Ch10: 24,818 kb	Confirmation of c2-SSR
AU223548SSR	ACCACCACTGCAGAGACTCA	GACGCACCCATTCATCTTTT	Ch10: 26,353 kb	Confirmation of c2-SSR
CH05c07	TGATGCATTAGGGCTTGTACTT	GGGATGCATTGCTAAATAGGAT	Ch09: 12,363 kb	Confirmation of c3-SSR
Hi05e07	CCCAAGTCCCTATCCCTCTC	GTTTATGGTGATGGTGTGAACGTG	Ch09: 14,303 kb	Confirmation of c3-SSR
13C2_30348-HRM	TACTTTAGCACCACCTTGTT	TGCCCGTTTAGTATATCACC	Ch09: 15,681,366	Confirmation of c3-HRM
C4935	TTTCCCAGCTGAAAAACTCG	GCAGAGAAATCCGCAGAAAC	Ch09: 17,787 kb	Confirmation of c3-SSR
CH04c07	GGCCTTCCATGTCTCAGAAG	CCTCATGCCCTCCACTAACA	Ch14: 24,205 kb	Confirmation of c4-SSR
C1374	CGGATCACAGACGCCAT	GCGTCATTTCAACAGCTTCA	Ch14: 24,421 kb	Confirmation of c4-SSR
C14087	CACCGCGTCAAAAATACCTT	CTTGTTGTTTCCCTCCCAAA	Ch06: 4,908 kb	Confirmation of c5-SSR
Hi08g03	ATTCACTTCCACCGCCATAG	GTTTGGAATGATTGCGAGTGAAGC	ch06: 6,119 kb	Confirmation of c5-SSR
CH03d07	CAAATCAATGCAAAACTGTCA	GGCTTCTGGCCATGATTTTA	ch06: 8,113 kb	Confirmation of c5-SSR
CH01h10	TGCAAAGATAGGTAGATATATGCCA	AGGAGGGATTGTTTGTGCAC	Ch08: 27,444 kb	Confirmation of c6-SSR
C13470	TCGATTCCTCAATCTCTCTCA	ATCGGAGAAAACCCAAATCC	Ch08: 30,478 kb	Confirmation of c6-SSR
MD10G1185400-Co	ATGGAGACATTAGATCAGAATCTTGT	CCATGATTGAAGACCTGGAAAAATCCG		qRT-PCR
MD17G1272100	GAGCCATCTTCCTGGGATT	CCCACCATGCATTCACTTT		qRT-PCR
MD07G1306900	TAAATGTGGAGGGAGGAGTTTT	TCTGAATTTCCTCCCACTTTCT		qRT-PCR
MD04G1096200	AGCGATTTTCGCTGAAGTG	TCAATCTGTCCAGGGTGGT		qRT-PCR
MD08G1025800	AGCACCTGGACGATCTGAC	TGCTGGGTGGTGATGTTTAT		qRT-PCR
MD01G1001600-Actin	GGCTGGATTTGCTGGTGATG	TGCTCACTATGCCGTGCTCA		qRT-PCR

Table S6. RNA-seq samples and statistics

Progeny #/ Sample name	Co genotype	Phenotype	Mapped reads		unique reads		non-specifically		Unmapped reads		Total reads		Raw reads	Removed raw reads
			Count	%	Count	%	Count	%	Count	%	Count	%	Count	%
2230	*Coco*	Columnar	36,969,406	83.58	35,412,001	80.05	1,557,405	3.52	7,265,384	16.42	44,234,790	100	49,654,918	10.92
3213	*Coco*	Columnar	6,022,338	83.25	5,763,783	79.68	258,555	3.57	1,211,721	16.75	7,234,059	100	18,141,844	60.13
3214	*Coco*	Columnar	40,154,110	81.16	38,462,890	77.74	1,691,220	3.42	9,320,090	18.84	49,474,200	100	54,239,730	8.79
3233	*Coco*	Columnar	17,009,862	83.31	16,300,008	79.83	709,854	3.48	3,408,462	16.69	20,418,324	100	27,530,597	25.83
2220	*Coco*	Standard2	17,798,795	82.98	17,080,776	79.63	718,019	3.35	3,651,246	17.02	21,450,041	100	29,454,486	27.18
2223	*Coco*	Standard2	27,163,039	83.19	26,037,801	79.74	1,125,238	3.45	5,488,445	16.81	32,651,484	100	38,412,645	15.00
3253	*Coco*	Standard2	27,403,712	83.9	26,243,485	80.35	1,160,227	3.55	5,259,717	16.1	32,663,429	100	37,466,963	12.82
3258	*Coco*	Standard2	26,822,239	83.65	25,731,388	80.24	1,090,851	3.4	5,244,456	16.35	32,066,695	100	34,627,016	7.39
3221	*coco*	Standard1	23,042,707	85.08	22,075,512	81.51	967,195	3.57	4,041,226	14.92	27,083,933	100	33,713,293	19.66
3222	*coco*	Standard1	25,921,658	85.76	24,797,687	82.04	1,123,971	3.72	4,305,075	14.24	30,226,733	100	32,925,469	8.20
3227	*coco*	Standard1	12,731,457	85.05	12,207,680	81.55	523,777	3.5	2,237,741	14.95	14,969,198	100	21,123,725	29.14
3239	*coco*	Standard1	18,276,683	84.01	17,503,737	80.45	772,946	3.55	3,479,376	15.99	21,756,059	100	32,657,561	33.38
Sum			279,316,006		267,616,748		11,699,258		54,912,939		334,228,945		409,948,247	
Mean			23,276,334	83.7	22,301,396	80.2	974,938	3.5	4,576,078	16.3	27,852,412	100.0	34,162,354	21.5
SD			9,663,852	1.2	9,255,440	1.1	408,855	0.1	2,175,319	1.2	11,795,856		10,309,416	15.1

Table S7. Differentially expressed genes (DEGs) among the three phenotypes columnar, standard1 (Std1) and standard2 (Std2)

Table S8. Genes expressed under c2 and c3

Table S9. Differences and similarities in informative segregation types inferred for dominant and recessive traits

	Segregation types	Symbol	Source pools	Usefulness	Comments	Reference
Recessive	A	<hk × hk>	Common to both pools	Yes	Commonly used	This study
	B	<nn × np>	Common to both pools	Yes	Hidden	
	C	<lm × ll>	Common to both pools	Yes	Hidden	
	D	<pp × np>	Common to both pools	?	Hidden	
	E	<lm × mm>	Common to both pools	?	Hidden	
Dominant	I	<lm × mm>	Dominant trait specific pool	Yes	Commonly used	Dougherty et al 2018
	II	<lm × ll>	Common to both pools	Yes	Hidden	
	III	<hk × hk>	Common to both pools	Yes	Hidden	
